# A transcriptome analysis reveals a role for the indole GLS-linked auxin biosynthesis in secondary dormancy in rapeseed (*Brassica napus* L*.*)

**DOI:** 10.1186/s12870-019-1866-z

**Published:** 2019-06-18

**Authors:** Lei Liu, Fuxia Liu, Jinfang Chu, Xin Yi, Wenqi Fan, Tang Tang, Guimin Chen, Qiuhuan Guo, Xiangxiang Zhao

**Affiliations:** 1 Jiangsu Key Laboratory for Eco-agriculture Biotechnology around Hongze Lake, Huaiyin Normal University, Huai’an, 223300 China; 2 Jiangsu Collaborative Innovation Center of Regional Modern Agriculture and Environment Protection, Huaiyin Normal University, Huai’an, 223300 China; 3 School of Food Science and Engineering, Yangzhou University, Yangzhou, 225127 China; 4 National Centre for Plant Gene Research (Beijing), Institute of Genetics and Developmental Biology, Chinese Academy of Sciences, Beijing, China

**Keywords:** *Brassica napus*, Secondary dormancy, Germination, Volunteer plant, Phytohormone, RNA-seq analysis

## Abstract

**Background:**

*Brassica napus* L. has little or no primary dormancy, but exhibits great variation in secondary dormancy. Secondary dormancy potential in oilseed rape can lead to the emergence of volunteer plants that cause genetic contamination, reduced quality and biosafety issues. However, the mechanisms underlying secondary dormancy are poorly understood. In this study, cultivars Huaiyou-WSD-H2 (H) and Huaiyou-SSD-V1 (V), which exhibit low (approximately 5%) and high (approximately 95%) secondary dormancy rate, respectively, were identified. Four samples, before (Hb and Vb) and after (Ha and Va) secondary dormancy induction by polyethylene glycol (PEG), were collected to identify the candidate genes involved in secondary dormancy via comparative transcriptome profile analysis.

**Results:**

A total of 998 differentially expressed genes (DEGs), which are mainly involved in secondary metabolism, transcriptional regulation, protein modification and signaling pathways, were then detected. Among these DEGs, the expression levels of those involved in the sulfur-rich indole glucosinolate (GLS)-linked auxin biosynthesis pathway were markedly upregulated in the dormant seeds (Va), which were validated by qRT-PCR and subsequently confirmed via detection of altered concentrations of indole-3-acetic acid (IAA), IAA conjugates and precursors. Furthermore, exogenous IAA applications to cultivar H enhanced secondary dormancy.

**Conclusion:**

This study first (to our knowledge) elucidated that indole GLS-linked auxin biosynthesis is enhanced during secondary dormancy induced by PEG, which provides valuable information concerning secondary dormancy and expands the current understanding of the role of auxin in rapeseed.

**Electronic supplementary material:**

The online version of this article (10.1186/s12870-019-1866-z) contains supplementary material, which is available to authorized users.

## Background

Seed dormancy is an important adaptive trait of seed plants and is influenced by both genetic factors and environmental cues to ensure timely germination for propagation [1, 2]. The classification of primary and secondary dormancy is widely accepted based on the occurrence time. Primary dormancy, which has been thoroughly studied in model plants, is induced and maintained during seed development on the mother plant, and peaks when the seeds mature and are released by after-ripening [3]. By contrast, secondary dormancy, which is mainly responsible for dormancy cycling, is defined as the inability of mature and non-dormant seeds to germinate under adverse conditions (anoxia, high temperature and/or drought) [4, 5]. Once primary dormancy is released and adverse conditions start to take effect, secondary dormancy of dispersed seeds that originate from seed shedding in the field may be induced. Then the secondary dormant seeds involve repeated induction and termination in response to environmental conditions until the proper conditions for germination are met [6].

*Brassica napus* L. is one of the most important cultivated oilseed crops worldwide. Oilseed rape seeds undergo little or no primary dormancy [7, 8], but exhibit different secondary dormancy potential [5, 9]. Before and during the harvest of the seeds, seed losses can reach up to 10,000 seeds per m^2^ because of silique shattering [10]. Under adverse conditions, the shed seeds can be induced into secondary dormancy and remain alive for 10 years or even longer, thereby increasing the size of the seed bank in the soil [11–13]. Once encountering favorable conditions, the dormant seeds will germinate to emerge as a weed (if among other crop species) or as volunteer plant (among oilseed rape crops) during subsequent crop cultivation. The volunteer plants might cross-pollinate with the subsequent crop plants and reduce seed quality [7, 14]. Moreover, the volunteers could lead to biosafety issues by cross pollination given the persistence of genetically modified oilseed rape in the seed bank [15, 16]. In this situation, genetic improvement resulting in plants with low or no secondary dormancy will help efficiently reduce the soil seed bank to avoid biosafety risks. Therefore, research on the genetic basis and mechanism underlying secondary dormancy is of great theoretical and practical significance.

Oilseed rape shows large and significant variation in its capacity for secondary dormancy (< 60% for winter varieties, < 80% for spring varieties), and the trait is highly heritable [8, 10, 16, 17]. The contributions of genotype, seed size and harvest time to secondary dormancy have been quantified and the genotype contribution ranged from 44 to 82% of the total variation [7], suggesting that genotype was the decisive factor in secondary dormancy. With PEG6000 treatment for 2 weeks in the dark [5, 10, 18], four quantitative trait loci (QTL) for secondary dormancy located on chromosomes A5, C3, C5 and C8 were detected via a doubled haploid (DH) population derived from a cross between the cultivar Express 617 (30.4% secondary dormancy) and the accession R53 (61.7% secondary dormancy), which explained 35% of the phenotypic variation [16].

Previous studies have indicated that the molecular mechanism underlying primary dormancy relies on the signal integration of both hormone (abscisic acid (ABA) / gibberellic acid (GA) balance) and gene expression (*DOG1*) thresholds [19–21]. ABA and GA have been recognized as the predominant hormones that antagonistically regulate seed dormancy [22]. Recently, increasing evidence have shown that another phytohormone, auxin, is also crucial for primary dormancy in *Arabidopsis*, although its effects are somewhat debatable [23–26]. ABA plays crucial roles in rapeseed secondary dormancy [7]; however, whether auxin plays pivotal roles remains unknown. Furthermore, the mechanism by which secondary dormancy is induced and maintained is poorly understood.

RNA sequencing (RNA-seq) has become a very useful tool for understanding the relationships between the underlying mechanism of developmental processes and gene expression profiles at the whole-genome level in *Brassica napus*, including the genetic basis of salt tolerance [27], that of root length [28], and the effects of night temperature on storage lipids [29]. In the present study, we performed RNA-seq using two representative cultivars (approximately 95 and 5% secondary dormancy, respectively) to detect the differentially expressed genes (DEGs) responsible for secondary dormancy. This study aimed to determine the significant transcriptome changes in secondary dormant and non-dormant seeds and revealed a role of indole glucosinolate (GLS)-linked auxin biosynthesis in secondary dormancy.

## Methods

### Plant materials

Two cultivars of winter type rapeseed (*Brassica napus* L.) were used in this study, showing significant difference in secondary dormancy potential. The low secondary dormancy cultivar namely Huaiyou-WSD-H2 (abbreviated as H) showed secondary dormancy rate lower than 5% induced by PEG and the high secondary dormancy cultivar namely Huaiyou-SSD-V1 (abbreviated as V) showed secondary dormancy higher than 90% (Table 1) induced by PEG. Besides, the two selected cultivars are also different in many seed quality traits (Additional file 2: Table S12). Both cultivars were screened from those of advanced generations bred by Dr. Zhao’s research group in Huaiyin Normal University.

**Table 1:** Statistical analyses of secondary dormancy ratio of cultivars H and V from three independent replicates

Year	Cv.	Germination in water (Control)					Secondary dormancy after PEG induction												
		No. of germinated seeds				Rate (%)	No. of non-germinated seeds				No. of germinated seeds after dormancy release				Secondary dormancy rate (%)				
		I	II	III	IV	Average	I	II	III	IV	I	II	III	IV	I	II	III	IV	Average^a^
2015	H	100	100	100	100	100	4	5	2	3	4	5	2	3	4	5	2	3	3.5 ± 1.3
	V	100	100	100	100	100	92	91	96	95	92	91	96	95	92	91	96	95	93.5 ± 2.4
2016	H	100	100	100	100	100	0	2	4	1	0	2	4	1	0	2	4	1	1.8 ± 1.7
	V	100	100	100	100	100	100	99	97	95	100	99	97	95	100	99	97	95	97.8 ± 2.2
2017	H	100	100	100	100	100	4	3	3	3	4	3	3	3	4	3	3	3	3.3 ± 0.5
	V	100	100	100	100	100	94	95	95	98	94	95	95	98	94	95	95	98	95.5 ± 1.7

H represents the cultivar Huaiyou-WSD-H2, and V denotes the cultivar Huaiyou-SSD-V1

Cv. represents cultivar

I, II, III and IV denote the number of four dishes in one biological replicate, respectively

^a^Data are shown as mean ± SD

### Induction of secondary dormancy

The rapeseed cultivars H and V were planted in the experimental field in Biotechnology Park of Huaiyin Normal University, with each cultivar forming a plot of 30 m^2^. Individual plants of each cultivar were bagged separately for self-pollination at flowering stage. After maturation the selfed seeds of each cultivar were collected, dried and stored under constant temperatures at 25 °C for secondary dormancy testing.

Secondary seed dormancy was induced in accordance with the protocol described by Gruber et al. [17] with slight modifications. Dry seeds of H (4 × 100) and V (4 × 100) were placed in four Petri dishes (100 seeds per Petri dish) layered with two pieces of filter paper for one biological replicate. Then 8 ml of PEG6000 solution were added to the dishes with an initial osmotic potential of − 1.5 MPa and put into a cardboard box. The cardboard boxes were then wrapped with black cloth and placed in a growth chamber (MLR-351H, SANYO) in the dark at 20 °C for two weeks. Afterward, the seeds were washed with water under green light (500-600 nm) to remove any PEG and immediately transferred into new Petri dishes supplemented with 6 ml of pure water. Then the Petri dishes were put back to the card boxes with black cloth and transferred to the chamber for germination. Eight days later, the germinated seeds were removed and the remaining seeds were placed in chamber under alternating light and temperature conditions (12 h dark at 3 °C and 12 h light at 30 °C) to release dormancy to test the seed viability. The germinated seeds under the alternating conditions were considered to be secondary dormant seeds and the rest were unviable. Secondary dormancy rate was calculated as: Secondary dormancy (%) = secondary dormant seeds *100 / (100-unviable seeds). Simultaneously, both stains H and V imbibed in water instead of PEG were used as a control.

Secondary dormancy rates of both cultivars H and V were calculated in the consecutive three years from year 2015 to 2017.

### Sample harvest and RNA extraction

For one biological replicate, four samples, including mature seeds of cultivar H and V (Hb and Vb) and PEG6000-induced seeds (Ha and Va), were collected. The details per replicate are as follows: During secondary dormancy induction, seeds of H and V were imbibed in PEG6000 with an initial osmotic potential of − 1.5 MPa for two weeks. At that time, seeds of H, which were not induced into secondary dormancy, were sampled and referred to Ha. Seeds of V were washed with water under green light to remove any PEG and immediately transferred into new Petri dishes supplemented with 6 ml of pure water to germinate in the dark for another 8 days. Afterwards, the germinated seeds were removed and the remaining seeds induced into secondary dormancy were sampled and referred to Va. Meantime, mature seeds of H and V, which were not subjected to PEG treatment, were sampled and referred to Hb and Vb respectively. Three independent replicates were conducted and 12 samples in total were harvested for RNA extraction.

The total RNA was extracted from 0.1 g of mature seeds per sample using an Aidlab RN38 RNA extraction kit (Aidlab, China) according to the manufacturer’s instructions with slight modifications by adding 300 μl chloroform to centrifuge before the second step.

### RNA-seq experiments

The quality of the total RNA was analysed using an Agilent 2100 Bioanalyzer with threshold value of RNA integrity number (RIN) ≥ 7.5. The RNA (0.1–0.4 μg) was then precisely quantified using a QUBIT RNA Assay Kit (Invitrogen, America). mRNA was purified from the total RNA using magnetic beads with oligo (dT) primers and then fragmented into 120–210 bp. First-strand cDNA was synthesized via a SuperScript II Kit (Invitrogen, America) and the second strand was synthesized by DNA polymerase I. After end repair and 3′ end adenylation, the indexed adapter was ligated to both ends using T4 DNA ligase. The cDNA was then purified twice by AMPure XP Beads (Beckman Coulter, America) to eliminate redundant adapters and amplified via PCR. Finally, a gel purification procedure was performed to select the fragments ranging from 300 to 350 bp in size to produce a paired-end library. The fragment size was controlled via an Agilent 2100 Bioanalyzer, and the yield was assessed via a KAPA qPCR Kit (Kapa Biosystems, America). A 10 μl library (2 nM) was fixed onto cBot (Illumina, America) and sequenced on an Illumina X10 platform with 150 bp paired-end reads by CapitalBio Corporation, Beijing, China.

### RNA-Seq data processing and gene expression annotation

RNA-seq raw reads were processed using an NGS-QC toolkit [30], after which low-quality and adapter-contaminated sequences were discarded. The paired high-quality reads were then aligned with the reference genome of *Brassica napus* (http://www.genoscope.cns.fr/brassicanapus/data/) via HISAT [31]. To obtain more functional annotations, all the detected genes were blasted against the *Arabidopsis* genome (The *Arabidopsis* Information Resource (TAIR), http://www.arabidopsis.org/Blast/index.jsp) using BLASTN. The functions of the unigenes were annotated via BLASTX searches against the non-redundant (NR), SwissProt, and Uniprot databases (E < 1e-5). The Gene Ontology (GO) annotations were determined using Blast2GO with the UniProtKB/SwissProt database. Pathway annotations were performed to understand gene functions via BLASTX of the Kyoto Encyclopedia of Genes and Genomes (KEGG) database. For GO and KEGG enrichment, a corrected *p* ≤ 0.05 value was set as the threshold. The MapMan software (http://mapman.gabipd.org/home, 19.11.2010) was used to provide a graphical overview of the metabolic and regulatory pathways.

### Quantification of gene expression levels and analysis of DEGs

Gene expression levels were calculated using the fragments per kilobase per million fragments (FPKM) method [32] via Cuffquant [33] and Cuffnorm softwares [33]. Differential gene expression analysis was carried out by Cuffdiff [34] software based on two criteria: (a) an absolute of log_2_ fold-change (FC) ≥ 1 and (b) a q value (false discovery rate (FDR)) ≤ 0.001.

### Real-time qRT-PCR analysis of DEGs

To validate the DEGs identified from RNA-seq analysis, real-time qRT-PCR was conducted. The total RNA was isolated from newly treated samples (not those used for RNA-seq). 1 μg of total RNA free of DNA contamination was used to synthesize first-strand cDNA, which was synthesized with oligo (dT) primers via a the Revert Aid First Strand cDNA Synthesis Kit (K1622, Thermo). Considering the allopolyploid oilseed rape genome, we download all loci sequences of the same gene which is to be detected according to their accession numbers (http://brassicadb.org/brad/datasets/pub/Genomes/Brassica_napus/). Gene-specific primers were designed according to 1) the same sequences from all loci of the same gene and 2) unique sequences to the gene (http://bioinfo.ut.ee/primer3/; Table 2). The expression was measured from all loci of the same gene. PCR products were confirmed to be specific by melt curve analysis and be a single band by gel electrophoresis. The reaction mixtures were prepared with a SYBR PrimeScript™ RT-PCR Kit (RR820A, TaKaRa) and then loaded into a Bio-Rad CFX Manager instrument. The PCR procedure was as follows: 95 °C for 5 s followed by 40 cycles of 95 °C for 3 s and 60 °C for 30 s. Three biological replicates per sample and three technical replicates were conducted, and the relative expression levels were calculated using the 2^-△△CT^ method [35]. As an internal control, *BnaCAT1* (BanC07g15270D) was used to normalize the gene expression (Additional file 2: Table S3).

**Table 2:** Gene ID and gene-specific primers for qRT-PCR validation

Gene ID	Forward primer	Reverse primer	*Arabidopsis* homolog
BnaA03g01690D	GCAGGAGACATATTCCAGATTGTG	ATCGACCAACATGATGTGCTCAG	ASA1
BnaC08g25400D	CCAACAACACCAACAGAGAGAATG	GACTTTGTGAGACTCTCAAGCTCC	TSA1
BnaA10g09090D	CTCACTCCATCAGTGCAGGATTG	GTTCAACACGACTCTGCTTCCG	TSB1
BnaA01g34610D	CTCAACGAGTGCTCCGAAGTTAC	GACCATAACCAACGGTTTACACAG	CYP79B2
BnaA08g04520D	GAGAAACCATAGCAGACGCGAAG	CTCTACCATTGCGACTCCAAGAT	CYP83B1
BnaA07g31260D	GTAAAGGCTTGTCTGTGTACGGTC	CGTCTCTTCCACTAACCCATCG	SOT16
BnaA04g17910D	CCTCACACTCCACATCATGTTC	TCAAGTGTCTCCTTCCAAGCAG	GSTF9
BnaA07g00460D	GTCGATTTGGTGTGTGATAGGC	CCTCAAGCATATGAGCTTCCAC	SUR1
BnaA03g57920D	TGAGAGAGAGATGGGTCGTGTTC	GCTTCACAATGGACTCACCAAC	IGPs
BnaA02g05170D	GCTGTGGATTTAGGACAGATTGAA	CTGGAACAGGCAAGTCAAAGCTC	AAO1
BnaC04g19270D	GGAGGGATTGAGAATTTGTTGAA	GCGAATCGACATCTCTATGCTC	APR1
BnaC01g00790D	GGTGAAGTGGCAAAGCTGTTTG	GATGATGTTTTCAGCCATCTGAC	APK2
BnaA03g59800D	GAGGGATTGAGAATCTGTTGAAGC	ATAAGCGAATCCACATCTCTATGC	APR2
BnaA10g11280D	GACGGTTGCTCACGAAACTCTC	ACTAAGCATTGTGTTGCTCTCTCC	CYP81F2
BnaA01g12900D	CATCTGACGACAATATTCCCTGAG	CACATGCTTCAACAAACCATCTCT	GSH1
BnaC02g40130D	ACGTGGTGAATTTCTTGGTCCAC	GTTGACACCCTTCTCCTTGATGAC	TGG2
BnaCnng66020D	ACAAACGGTGGGAGTGGTAA	GTGATCACCGCTCGCATATC	WRKY33
BnaC04g38910D	TGACGGATACAGGTGGAGGAAG	GTCAAGATGTGGTGGAAGTTATCG	WRKY45
BnaA05g28580D	CAGTGATGAACGCAAGATCATC	AAGCCTCCTCTCTCAGACACAAC	SZF1
BnaAnng08970D	TTCTCCAACTCGTCCAGGC	GGCACTTGTCGTAGTTTCCG	GASA1
BnaA09g44500D	CAAGAAGATGGAGAAGTCGGCTAG	CATGAGAAGATTCGAGGTTGGTG	MYB51
BnaC04g38910D	ACGACGGAGCACTACGTCACTATC	ATCACCAGCAGTATAAGCAGAAGC	Bzip44
BnaCnng74400D	GTATTGGATTCAGAAGTGCCTGG	TCTGTGAGATGCACAAATGCTG	TRN1
BnaC01g12390D	CTGATAGTCTCAACACCAAACTTCG	GTCCTTCTGCTACACTCTCAACAGC	HEN1
BnaC03g14590D	ACGAGCTAACCGAAGCTGATG	AAACATTCCAGCACCAAATGTGT	FQR1
BnaA09g05810D	TGCAGATGGTCTTAAGAGCTACCAG	TTCCTTCTTCTCCGACTCATCTCC	CNX1
BnaA09g52180D	ATCCACAAGACATCATGGCTGAG	CTGGTTCATCTTCAATTCCACATTC	CATHB3
BnaC03g30870D	GCCTTTGAAGATGGAGACCTCA	TCGGTGAAGAGCTTCTTGGTG	GSTF2

### Analysis of the content of auxin intermediates

Analysis of IAA, tryptophan, IAA-Asp, and IAA-Glu:

Samples for IAA, tryptophan, IAA-Asp, and IAA-Glu analysis were extracted and purified as described in the method reported by Fu et al. [36] with some modifications: Dry seeds or seeds subjected to PEG6000 treatment (200 mg, DW) were ground to fine powder in liquid nitrogen, then extracted with 80% MeOH containing internal standards (10 ng ^13^C_11_, 10 ng ^15^N_2_-trp and 2.5 ng ^2^H_2_-IAA) and at − 20 °C for 16 h. After centrifugation, the supernatant was collected and evaporated to dryness. The dried residue was reconstituted in 5% NH_4_OH, and loaded onto the Oasis MAX (Waters) cartridge. The cartridge was sequentially washed with 5% NH_4_OH, water and MeOH, and then was eluted with 5% formic acid (FA) in MeOH. The eluate was evaporated and re-dissolved in 80% MeOH for LC-MS/MS analysis. LC-MS/MS analysis was performed on a UPLC system (Waters) coupled to the 6500 Qtrap system (AB SCIEX). LC separation performed a BEH C18 column (1.7 mm, 2.1 × 100 mm; Waters) with mobile phase 0.1% FA (A) and acetonitrile (ACN) (B). The gradient was set with initial 2% B and increased to 50% B within 6 min at a flow rate of 0.3 mL/min. Tryptophan, IAA, IAA-Asp, and IAA-Glu were detected in positive ionization and MRM mode with transitions 205/188, 176/130, 291/130, and 305/130, respectively. ^2^H_2_-IAA was used as an internal standard to quantify IAA, IAA-Asp, and IAA-Glu, and ^13^C_11_,^15^N_2_-trp was used to quantify tryptophan level.

Analysis of indole-3-pyruvic acid (IPyA):

IPyA analysis was performed as described by Mashiguchi et al. [37] with slight modifications: About 100 mg of seeds was ground to fine powder in liquid nitrogen and was homogenized in methanol. The extracts were centrifuged and the supernatant was transferred to a glass tube. Three extractions were done quickly and the supernatants were combined. 2,4-dinitropjenylhydrazine (DNPH) and 1 N HCl were added to the extract and reacted overnight at 37 °C. The amount of DNPH added to the extract was 1 μg DNPH per 1 mg fresh weight sample. After precolumn derivatization, distilled water was added to the same volume of extract and then loaded onto an Oasis MCX cartridge (Waters). The cartridge was washed with 50% methanol and eluted with the 80% MeOH, and the eluate was evaporated to dryness. Finally, the dried DNPH-IPA was methylated with diethyl ether and diazomethane at room temperature to get DM-IPA. DM-IPA was dissolved into 50% acetonitrile for UPLC-MS/MS analysis.LC-MS/MS analysis was performed on a UPLC system (Waters) coupled to the 6500 Qtrap system (AB SCIEX). LC separation used a BEH C18 column (1.7 mm, 2.1 × 100 mm; Waters) with mobile phase 0.05% acetic acid (A) and 0.05% acetic acid/ACN (B). The gradient was set with initial 5% B and increased to 60% B within 8 min at a flow rate of 0.3 mL/min, then increased to 95% B for 13 min. DM-IPA was detected in negative ionization and MRM mode with transition 396/152. The level of IPyA was quantitatively analyzed by an external standard curve.

### Germination assays of exogenous auxin treatment

Germination assays were performed by placing rape seeds in Petri dishes lined with two layers of filter paper soaked in water or IAA solution (10 mg/L, 100 mg/L, 500 mg/L) for the indicated time at 20 °C in the dark, respectively. The IAA (I2886, Sigma) solution was first dissolved in ethanol and then in water to make the final concentration (10 mg/L, 100 mg/L, 500 mg/L). A seed was considered as germinated when its radical protruded through the seed coat. The non-germinated seeds after 8 days of imbibition were recorded to calculate the secondary dormancy ratio. Results from three independent replicates were obtained.

## Results

### Characterization of secondary dormancy potential in cultivars H and V

**Fig. 1:**
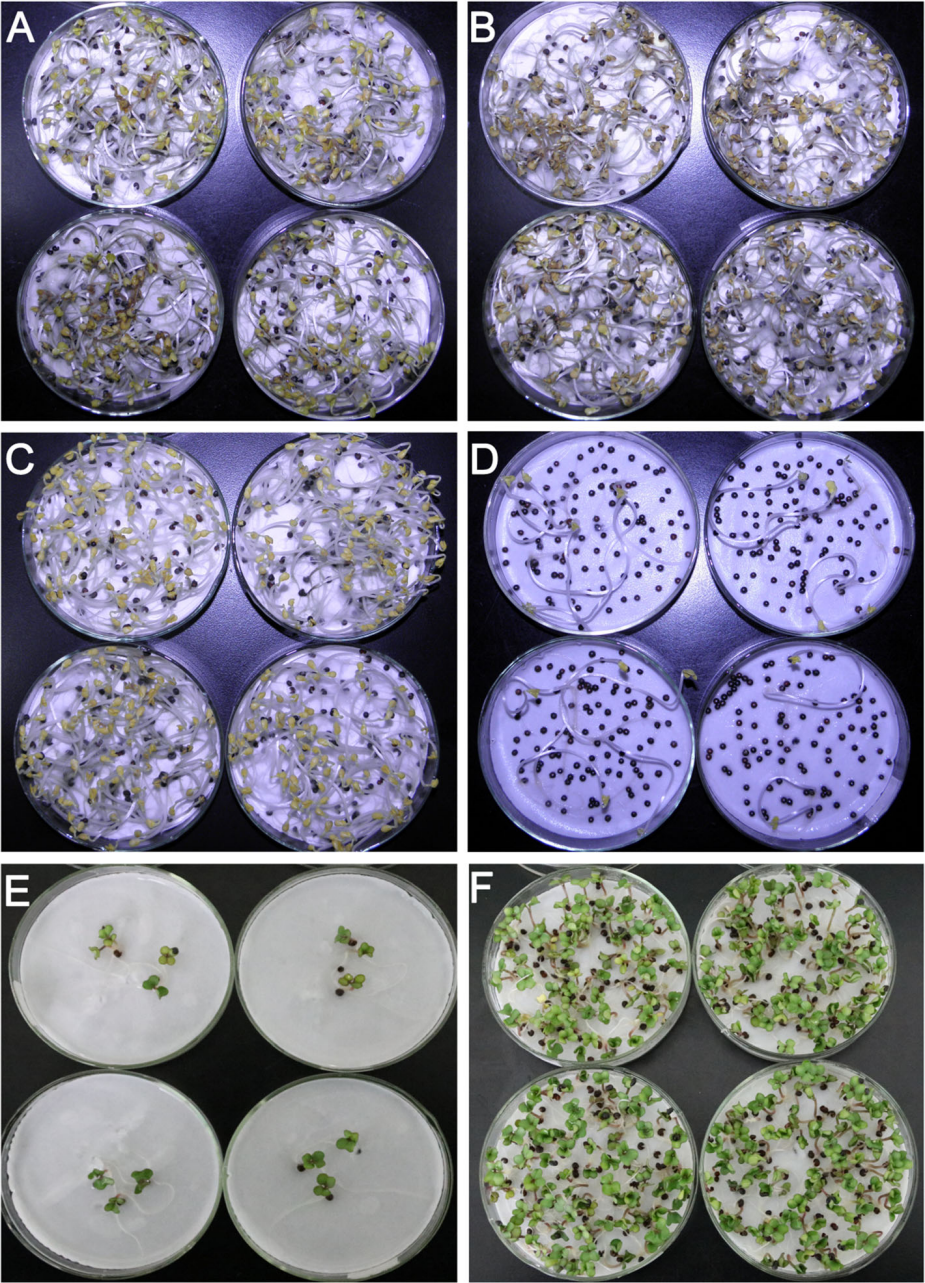
Characterization of the secondary dormancy phenotypes of cultivars H and V. (**a** and **b**) Images of seed germination assay of cultivars H (**a**) and V (**b**) in pure water in the dark for two weeks, respectively. (**c** and **d**) Secondary dormancy phenotypes of cultivars H (**c**) and V (**d**) following PEG6000 treatment for two weeks and incubation in water for another 8 days in the dark, respectively. (**e** and **f**) Secondary dormancy release assay of cultivars H (**e**) and V (**f**) following alternating light/temperature conditions, respectively

To isolate the candidate genes underlying secondary dormancy in rapeseed, two advanced generation cultivars, namely Huaiyou-WSD-H2 (abbreviated as H hereinafter) and Huaiyou-SSD-V1 (abbreviated as V hereinafter), were identified as the low and high representative genotypes, respectively. To test the seed viability, seeds of both H (Fig. 1a) and V (Fig. 1b) totally germinated in pure water in the dark at 20 °C, suggesting that all the seeds were viable. Following secondary dormancy induction via PEG treatment, almost all of the seeds of cultivar H germinated in pure water (Fig. 1c), while only few of the seeds of cultivar V germinated (Fig. 1d). To test whether the non-germinated seeds were secondary dormant (viable), secondary dormancy was broken under alternating light/temperature conditions. After dormancy release treatment, all the non-germinated seeds from H (Fig. 1e) and V (Fig. 1f) germinated, suggesting the non-germinated seeds were viable and secondary dormant. Statistical analyses of the secondary dormancy rate from year 2015 to 2017 revealed that cultivar H exhibited approximately 5% secondary dormancy potential and cultivar V exhibited approximately 95% secondary dormancy potential (Table 1).

### Analysis of RNA-seq quality control

To explore the transcriptomic changes during secondary dormancy induction, the representative cultivars H and V, which exhibit low (approximately 5%) and high (approximately 95%) secondary dormancy potential respectively, were used to perform RNA-seq experiments. The total RNA isolated from mature seeds (named Hb and Vb) and PEG-induced seeds (named Ha and Va) was qualified via Agilent 2100 analyzer and all of the RIN values were ≥ 7.9 (Additional file 1: Figure S1). A total of 12 cDNA libraries from three independent biological replicates were separately constructed using polyA^+^ RNA. The quality of RNA-seq experiments relies on the read coverage with respect to the reference genome of oilseed rape and the correlations between biological replicates [29]. The Phred quality score of > 30% (Q > 30%) was > 90% (Additional file 2: Table S1), suggesting that the sequencing was of high quality. In addition, approximately 75–82% of the clean reads were successfully mapped to the reference genome using TopHat2, and 66–75% of the clean reads were blasted to the unique genomic locations (Additional file 2: Table S1). As shown in (Additional file 1: Figure S2), an average of 60% of the total reads shared > 80% similarity with the reference genome. Furthermore, the correlations between the repetitions for each sample were > 0.90 (Additional file 1: Figure S3; Additional file 2: Table S2). These results collectively suggest that the RNA-seq data used in this study were highly reliable.

### Characterization of DEGs in secondary dormancy induction

**Fig. 2:**
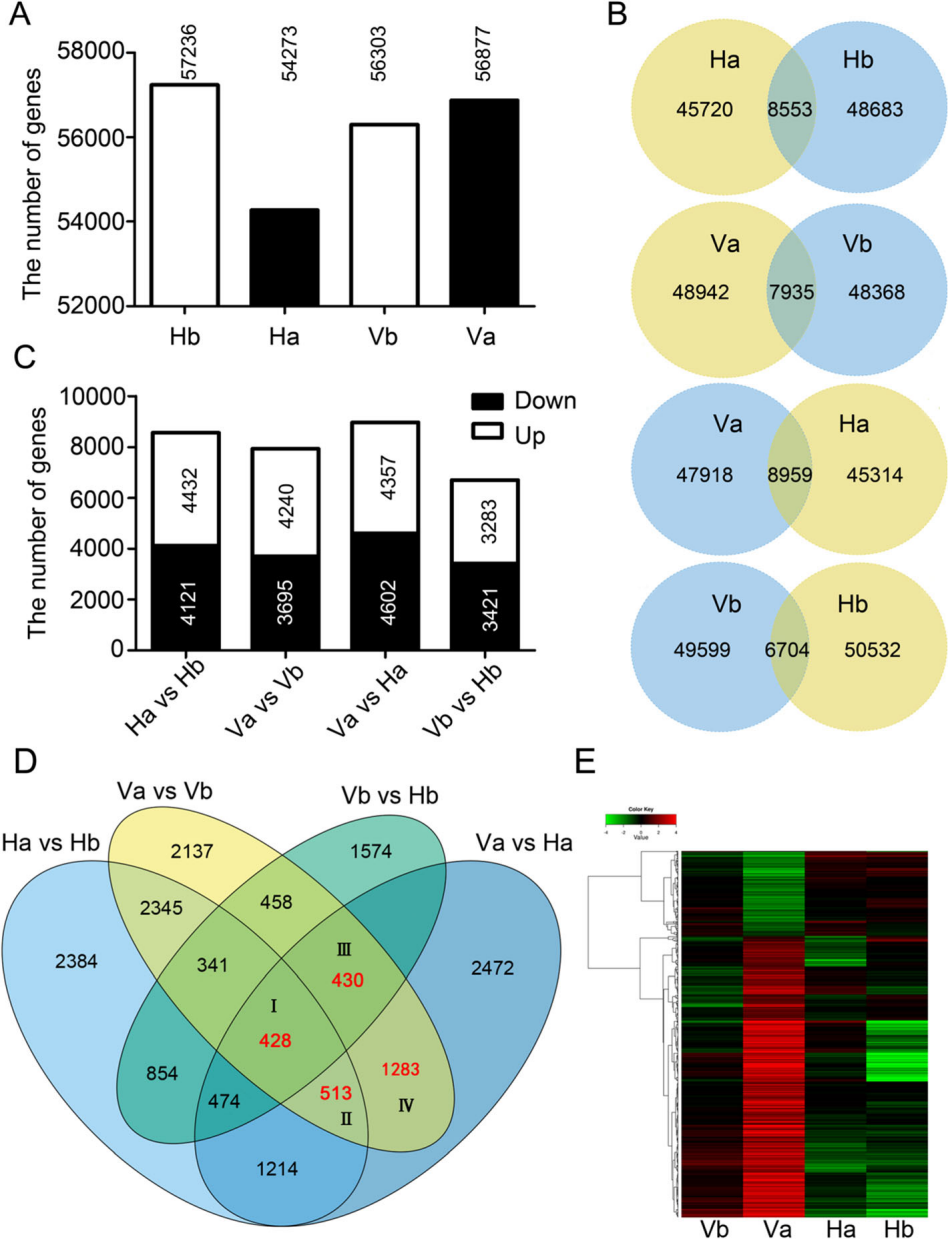
Identification and expression analysis of the DEGs involved in secondary dormancy. **a** Comparisons of the expressed gene numbers among the individual samples. The number above the bars indicates the gene number detected in the corresponding sample. **b** Venn diagrams showing the numbers of expressed genes and DEGs from the four comparison groups. The numbers in the intersections denote the DEGs in each group. **c** Comparisons of the number of DEGs among the four comparison groups and the number of up- and downregulated DEGs in the individual comparison groups. The numbers in the open and filled bars indicate the up- and downregulated DEGs numbers, respectively. **d** Venn diagram showing the DEGs between two individuals (Ha vs Hb, Va vs Vb, Vb vs Hb, Va vs Ha) and the candidate DEGs (in red) required for secondary dormancy in the first round of selection. **e** Hierarchical cluster analysis of 998 DEGs in which normalized FPKM values were used. The red colour denotes upregulated genes, and the green colour indicates downregulated genes, as shown in the above colour bar. The final normalized FPKM values range from − 4 to + 4

In Hb, Ha, Vb and Va, 57,236, 54,273, 54,501 and 53,386 genes were detected, respectively (Fig. 2a). In total, 66,703 genes accounting for 66.0% (66,703/101040) of the annotated genes in *Brassica napus* were expressed in the four samples. BLAST queries revealed that 50,589 of the detected 66,703 genes (75.8%) hit homologues in *Arabidopsis*, with an E < 10^− 5^ for nucleic acids. The detailed analyses of the detected genes including gene expression level, functional annotation, GO and KEGG analysis and MapMan analysis are shown (Additional file 1: Figure S4; Additional file 2: Table S3).

To determine the DEGs involved in secondary dormancy, four comparison settings including Ha vs Hb, Va vs Vb, Va vs Ha and Hb vs Vb, were analysed with a threshold of |log_2_(FC)| ≥ 1 and a FDR ≤ 0.001. Under these criteria, 8553 (4432 up and 4121 down), 7935 (4240 up and 3695 down), 8959 (4357 up and 4602 down) and 6704 (3283 up and 3421 down) genes were significantly differentially expressed in the Ha vs Hb, Va vs Vb, Va vs Ha and Hb vs Vb comparisons, respectively (Fig. 2b and c; Additional file 1: Figure S5). Much more DEGs were observed in Va vs Ha among the four comparison settings (Fig. 2b). Moreover, compared with downregulated genes, upregulated genes were identified more often in Ha vs Hb and especially in Va vs Vb (Fig. 2c). However, more downregulated genes were found both in both Va vs Ha and Hb vs Vb (Fig. 2c).

### Identification of candidate DEGs responsible for secondary dormancy

To identify the candidate genes associated with secondary dormancy, a Venn diagram containing 15 individual subgroups was constructed in conjunction with the four comparison settings (Fig. 2d). We focused predominately on the DEGs located in the intersection between Va vs Vb and Va vs Ha, which was divided into four subgroups (I-IV) that contained 428, 513, 430 and 1283 DEGs, respectively (Fig. 2d). Given that the DEGs in subgroups I and II also exhibited significant changes in Ha vs Hb, which showed non-dormant phenotypes, we collected mainly the inversely expressed genes (i.e., those upregulated in Va vs Vb but downregulated in Ha vs Hb and vice versa). For the DEGs in subgroups III and IV, a more stringent criterion, |log_2_(Va vs Vb)-log_2_(Ha vs Hb)| ≥ 1, was used to select the crucial candidate genes involved in secondary dormancy. On the basis of a second round of selection, 25, 44, 114 and 815 DEGs (998 in total) in the four subgroups were isolated, respectively (Additional file 2: Table S4). Among the 998 DEGs, 960 (96.2%) hit homologues in *Arabidopsis* and 674 (67.5%) were named and functionally annotated in *Arabidopsis* (Additional file 2: Table S4). To explore the expression profiles of the 998 DEGs on the basis of their normalized FPKM values, a heat map was constructed. As shown in Fig. 2e, the heat map clearly shows two clusters for the expression patterns of DEGs in the dormant sample (Va) and the number of bottom clusters (upregulated DEGs) was three times greater than that of the top clusters (downregulated DEGs). Taken together, these results indicated that the majority of candidate DEGs were significantly upregulated in secondary dormancy induction.

### Functional characterization of DEGs

To obtain the functional information of the DEGs, GO subcategory and KEGG pathway analyses were conducted. GO subcategory analysis revealed that “sulfur compound metabolism” and “glucosinolate metabolism” were highly enriched in upregulated DEGs (Table 3; Additional file 2: Table S5). Interestingly, the highly similar representative KEGG pathways “sulfur metabolism”, “glucosinolate biosynthesis” and “tryptophan metabolism” were also clearly enriched in upregulated DEGs (Table 3; Additional file 2: Table S6). In addition, the KEGG pathways identified for the DEGs in the first round of selection also suggested that the above three terms were highly enriched (Additional file 1: Figure S6).

**Table 3:** Detailed results of GO subcategory and KEGG pathways analyses for DEGs identified via RNA-seq analysis

Top 10 of detailed GO subcategory analysis		Top 10 of KEGG pathways	
Downregulated DEGs	Upregulated DEGs	Downregulated DEGs	Upregulated DEGs
Protein storage vacuole	**Sulfur compound metabolism**	Sulfur relay system	**Sulfur metabolism**
Plastid thylakoid membrane	Glycosyl compound metabolism	Fatty acid degradation	Carbon metabolism
Oxidoreductase activity	Oxoacid metabolism	Peroxisome	Biosynthesis of amino acids
Intracellular protein transmembrane import	Organic acid metabolism	Cutin, suberine and wax biosynthesis	Citrate cycle (TCA cycle)
Thylakoid membrane	Carbohydrate derivative metabolism	Carotenoid biosynthesis	2-Oxocarboxylic acid metabolism
Photosynthetic membrane	**Secondary metabolic process**	Tyrosine metabolism	Cysteine and methionine metabolism
Cytoplasm	Response to cadmium ion	alpha-Linolenic acid metabolism	**Glucosinolate biosynthesis**
Seed maturation	S-glycoside metabolic process	Ascorbate and aldarate metabolism	**Tryptophan metabolism**
Fruit development	**Glucosinolate metabolism**	Mismatch repair	Glycine, serine and threonine metabolism
Intramolecular lyase activity	Response to metal ion	Fructose and mannose metabolism	Pyruvate metabolism

The enriched pathways in bold are linked with auxin biosynthesis

Furthermore, a metabolic map and regulatory map showing an overview of the DEGs between Va and Ha were constructed using MapMan software. With respect to the cellular metabolism visualization, the majority of the DEGs were involved in pathways including lipid metabolism, secondary metabolism, amino biosynthesis and sulfur metabolism (Additional file 1: Figure S7; Additional file 2: Table S7), which validated the results of the GO and KEGG enrichment analyses. In the regulatory visualization, most of the DEGs were mapped to “transcription factor” “protein modification” “protein degradation” and “phytohormone signalling” (Additional file 1: Figure S7; Additional file 2: Table S7). The detailed information showing all the DEGs corresponding to their MapMan functional categories is listed in Table S7. The combination of the MapMan, KEGG pathway and GO subcategory analyses indicated that the DEGs that were associated with sulfur metabolism, GLS metabolism and phytohormone signalling pathways play dominant roles in secondary dormancy.

### Validation of the DEGs involved in indole GLS-linked auxin biosynthesis in secondary dormancy

**Fig. 3:**
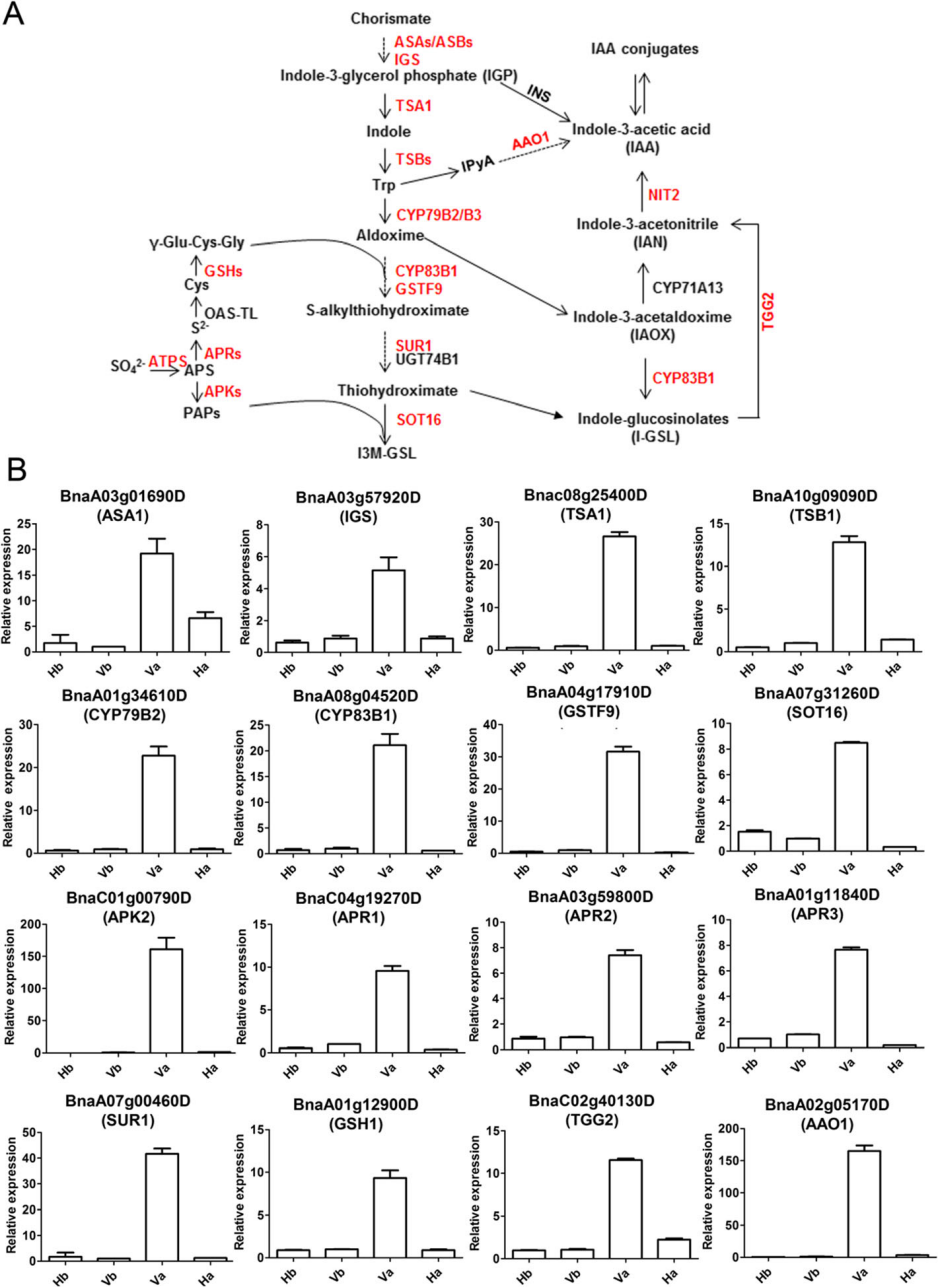
Validation of the DEGs involved in indole GLS-linked auxin biosynthesis via qRT-PCR. **a** The enriched metabolic pathway for the significant DEGs involved in secondary dormancy. The DEGs identified in the current study are marked in red. The genes in black were not detected in this study. The solid arrow indicates the direct promotion of the conversion, while the dotted arrow denotes the indirect promotion. **b** Verification of the enriched DEGs in (A) via qRT-PCR. Three independent biological replicates were tested and analysed. The relative expression levels of the DEGs were calculated via the 2^-ΔΔCT^ method. The corresponding gene name is shown in parentheses, as annotated by BLASTN in *Arabidopsis*. *BnaCAT1* was used as an internal control. Data are mean ± SD of three independent replicates

The pathways “tryptophan metabolism”, “sulfur metabolism”, “glucosinolate biosynthesis” and “auxin biosynthesis” are tightly linked (Fig. 3a). In this study, transcriptomic profiles revealed that the expression of the DEGs involved in tryptophan (Trp) biosynthesis in dormant Va seeds was markedly increased (Additional file 2: Table S8). Among the DEGs, *BnaA03g01690D*, encoding α-subunit of anthranilate synthase (*ASA1*) (Fig. 3a), showed about 20-fold and 4-fold upregulation in Va vs Vb and Va vs Ha, respectively (Fig. 3b). The expression of *BnaA03g57920D*, encoding indole-3-glycerol phosphate synthase (*IGS*) (Fig. 3a), exhibited a 6-fold increase in Va vs Ha (Fig. 3b). In addition, both Trp synthase α (*TSA1*) and β (*TSB1*) (Fig. 3a), encoded by *BnaC08g25400D* and *BnaA10g09090D*, respectively, were found to be upregulated more than 10 folds in Va vs Ha (Fig. 3b).

In this study, expression of the genes associated with the biosynthesis of sulfur donors was significantly upregulated in Va vs Vb and Va vs Ha (Additional file 2: Table S8). Indole GLS is biosynthesized from Trp (Fig. 3a). Interestingly, dramatic upregulation was observed only for the majority of indole GLS biosynthesis genes (Additional file 2: Table S8), while no change was observed for aliphatic or aromatic GLS genes (Additional file 2: Table S9). Furthermore, qRT-PCR validated that the expression of *BnaC01g00790D* (*APK2*) was upregulated more than 150-fold in Va vs Vb and Va vs Ha (Fig. 3b). In addition, the expression of *BnaA01g34610D* (*CYP79B2*), *BnaA08g04520D* (*CYP83B1*), *BnaA07g00460D* (*SUR1*) and *Bna04g17910D* (*GSTF9*), encoding the crucial enzymes required for the core structure formation of indole GLS, was upregulated 20-fold to 40-fold in Va vs Ha, as shown by qRT-PCR (Fig. 3b), which matched well with the RNA-seq results. In summary, contrast to that of the aliphatic and aromatic GLS biosynthesis genes, the expression of indole GLS biosynthesis genes was uniquely upregulated in secondary dormancy.

Notably, indole GLS is tightly linked with IAA biosynthesis [38–40]. IAA is biosynthesized from Trp-independent and Trp-dependent pathways [41, 42]. In the present study, expression of the Trp-independent IAA biosynthesis gene INS/TRPA1 showed no change in the dormant seeds (Additional file 2: Table S8). Correspondingly, in the Trp-dependent pathway, the most markedly changed DEG was *BnaA02g05170D* (*AAO1*), which was upregulated 150-fold in dormant seeds (Va) compared with the non-dormant seeds (Vb and Ha). Moreover, expression of the myrosinase-encoding gene *BnaC02g40130D* (*TGG2*) which catalyzes indole-GLS to produce IAN for IAA biosynthesis (Fig. 3a) was upregulated more than 5-fold in Va vs Ha (Fig. 3b). In addition to the strikingly upregulated biosynthesis genes, genes involved in auxin signaling transduction including *BnaC02g36370D* (*AFB2*), *BnaA10g27350D* (*SSR1*), *BnaA04g26870D* (*ABCB4*), *BnaA07g19210D* (*ABCB21*), *BnaC08g19040D* (*ARF5*) and *BnaA05g01310D* (*ARF11*) were also significantly changed in Va vs Ha (Table 4), thereby indicating a strong auxin signaling in secondary dormancy.

**Table 4:** List of DEGs involved in auxin transduction identified via RNA-seq analysis

Gene ID	*Arabidopsis* orthologs	Va vs Vb (log_2_FC)	Va vs Ha (log_2_FC)	Function description
BnaA07g28560D	IAR4	Up (1.7)	Up (1.9)	IAA conjugate sensitivity
BnaA09g42140D BnaC08g34560D	GH3.3	Down (−2.3/−3.7)	Down (−2.3/−3.3)	IAA-amido synthase activity
BnaC02g36370D	AFB2	Up (1.7)	Up (2.6)	The dominant auxin receptor
BnaC01g20800D	RMA2	Down (−1.5)	Down (−2.1)	Ubiquitinates Auxin binding protein 1
BnaA10g27350D	SSR1	Down (−1.7)	Down (−2.6)	Auxin polar transport
BnaC02g24140D	PILS3	Up (2.8)	Up (2.9)	Auxin efflux carrier
BnaA04g26870D	ABCB4	Up (5.2)	Up (2.8)	Auxin efflux transporter
BnaA07g19210D	ABCB21	Up (3.9)	Up (3.2)	Auxin efflux transporter
BnaC04g13950D	GSTU5	Up (2.3)	Up (2.9)	Response to auxin
BnaC05g50350D	PATL2	Up (3.9)	Up (2.1)	Response to auxin
BnaC05g23700D	PATL4	Up (1.2)	Up (1.8)	Response to auxin
BnaC08g19040D	ARF5	Up (2.3)	Up (2.9)	Auxin response factor
BnaA05g01310D	ARF11	Up (3.4)	Up (4.1)	Auxin response factor

### Determination of the metabolic differences in auxin intermediates

**Fig. 4:**
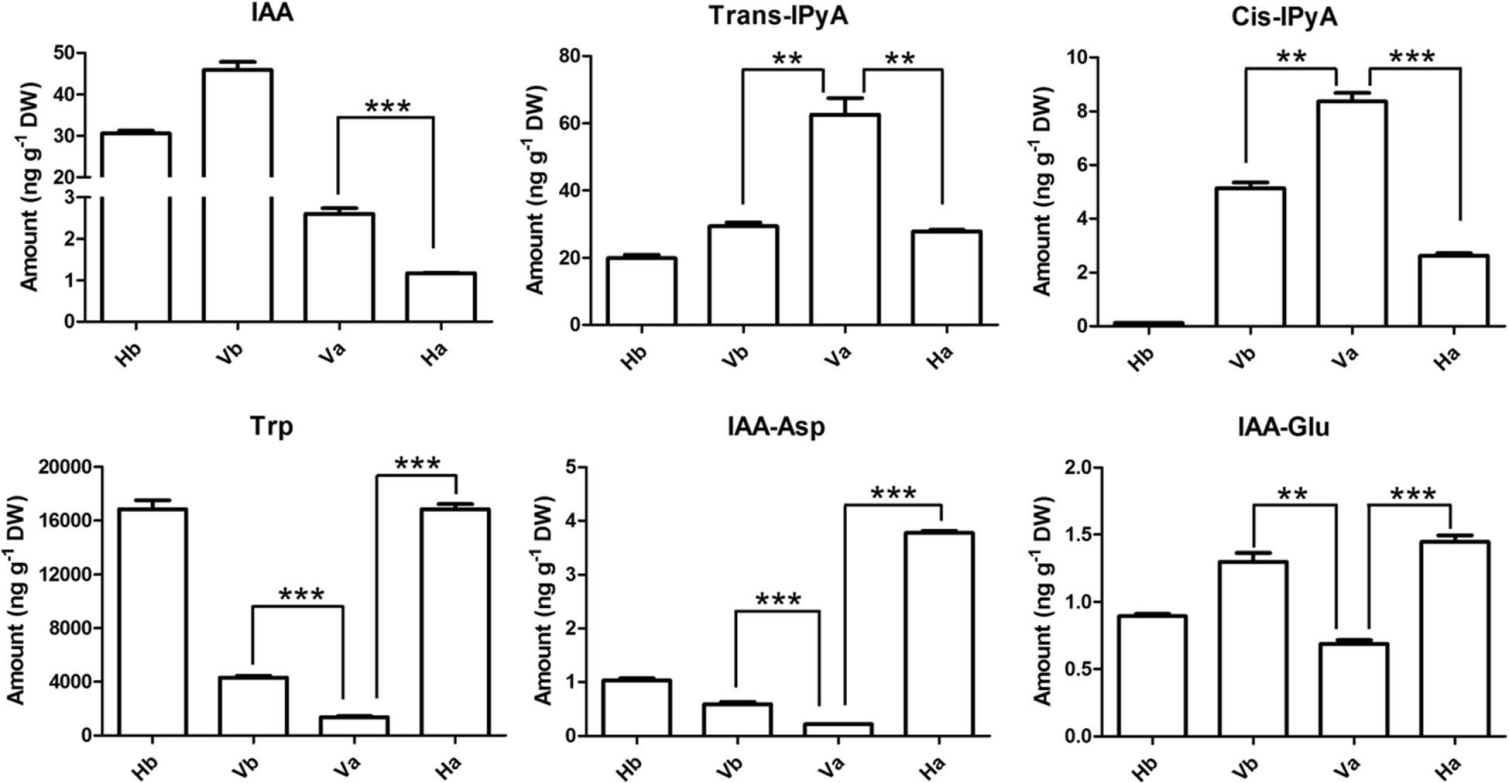
Measurements of IAA and its intermediates level. Free IAA, IAA precursors (IPyA and Trp) and IAA inactive conjugates (IAA-Asp and IAA-Glu) in dry seeds of H and V (Hb and Vb), and PEG-induced seeds of H and V (Ha and Va). IAA, indole-3-acetic acid; Trans-IPyA, *trans* indole-3-pyruvic acid; Cis-IPyA, *cis* indole-3-pyruvic acid; Trp, tryptophan; IAA-Asp, IAA-aspartate; IAA-Glu, IAA-glutamtate. The data from three independent replicates were normalized by seed dry weight (DW). Error bars ± SDs (*, *p* < 0.05; **, *p* < 0.01; ***, *p* < 0.001)

To investigate whether the activation of indole GLS-linked auxin biosynthesis led to metabolic differences, the concentration of auxin metabolites was determined. The level and distribution of auxin are tightly regulated by its synthesis (auxin precursors), inactivation (modified auxin forms) and transport [43]. Free IAA, main auxin precursors (IPyA), auxin substrates (Trp) and inactive IAA conjugates (IAA-aspartate (Asp) and IAA-glutamate (Glu)) were measured in the dry seeds and dormancy-induced seeds (Fig. 4). The level of free IAA was significantly higher in the dormant seeds (Va) than in the non-dormant seeds (Ha) (Fig. 4), in agreement with the upregulation of DEGs in the dormant seeds. Surprisingly, a dramatic decrease of free IAA was observed both in Ha vs Hb and Va vs Vb (Fig. 4), suggesting that the change was in response to PEG treatment rather than secondary dormancy. The concentration of IPyA, which is considered the predominant precursor for IAA biosynthesis [38], increased in the dormant seeds (Va) (Fig. 4). A negative feedback inhibition of Trp biosynthesis was released when Trp biosynthesis was impaired, leading to an increase in free IAA contents [44]. Similarly, a decrease in Trp concentration was observed in the dormant seeds (Va) (Fig. 4), resulting in the upregulation of Trp biosynthesis genes (Fig. 3b). Moreover, the concentrations of the inactive IAA conjugates (IAA-Asp and IAA-Glu) were much lower in the dormant seeds (Va) than in the non-dormant seeds (Ha and Vb) (Fig. 4). Taken together, these results indicate that indole GLS-linked auxin biosynthesis was enhanced in secondary seed dormancy.

### Exogenous IAA application promotes secondary dormancy in rapeseed

**Fig. 5:**
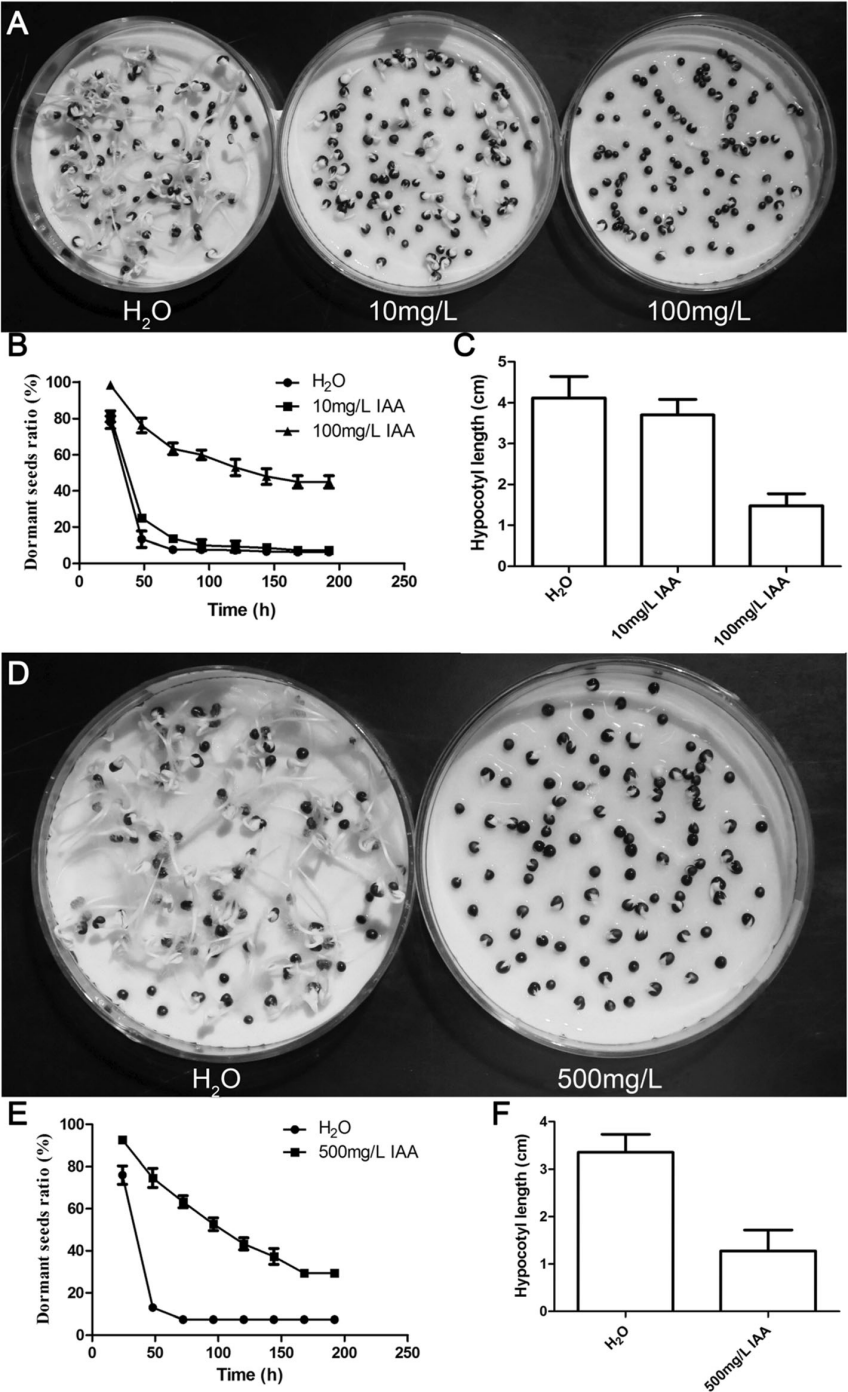
Effects of exogenous IAA treatment on secondary dormancy. Rape seed dormancy analysis of Ha supplemented with the indicated exogenous IAA solution for 8 days (**a**-**c**) and 1 day (**d**-**f**). (**a** and **d**) Images of secondary dormancy of Ha supplemented with the indicated exogenous IAA solution. (**b** and **e**) Statistics of dormant seed ratios at the indicated times. (**c** and **f**) Hypocotyl lengths of the geminated seeds grown in the dark for 8 days. The data shown are the means±SDs

To confirm the effects of auxin on secondary dormancy, exogenous IAA was applied to cultivar H. First, two strategies were applied to treat the low dormancy potential Hb in the dark: constant treatment with low concentrations of IAA (0 mg/L, 0.1 mg/L, 1 mg/L, 10 mg/L and 100 mg/L) (Additional file 1: Figure S8a) and treatment with high concentrations of IAA (0 mg/L, 100 mg/L, 500 mg/L, 1000 mg/L and 2000 mg/L) for 24 h (Additional file 1: Figure S8c). Under constant treatment conditions, the ratio of dormant seeds increased from 20 to 40% as the exogenous IAA concentration increased (Additional file 1: Figure S8b). When treated with 500 mg/L IAA for 24 h, the dormant ratio reached up to 80% (Additional file 1: Figure S8d). The same strategies were applied to treat the low dormancy sample Ha in the dark. Under conditions of constant application of 100 mg/L IAA, the dormancy ratio was 40% greater than that of the control (Fig. 5a and b), and compared with that in the control, the hypocotyl length in the 10 mg/L and 100 mg/L IAA treatments significantly decreased (Fig. 5c). Similarly, a 30% increase in the dormancy ratio and a 38% decrease in hypocotyl length were observed in response to the 500 mg/L IAA treatment for 24 h (Fig. 5d-f). Notably, low concentrations of IAA and the combination of ABA and IAA can’t mimic secondary dormancy (Additional file 1: Figure S9), in agreement with ABA treatment [12], but high concentration of IAA increased secondary dormancy ratio and elevated *BnaDOG1* expression level (Additional file 1: Figure S9), which is associated with secondary dormancy [4]. Collectively, the experimental results supported the main conclusion that high exogenous auxin enhanced secondary seed dormancy in the above conditions.

## Discussion

A long-standing question is whether secondary dormancy differs mechanistically and physiologically from primary dormancy [45]. Although extensively molecular and genetic analysis have been conducted on primary dormancy, very few researches uncovered the genetic basis underlying secondary dormancy in rapeseed [7, 16, 46]. In the previous researches, the secondary dormancy variation in AC Excel (80%) vs DH12075 (not shown) [7, 46] and in R53 (61.7%) vs Express 617 (30.4%) [16] was less significant than that of cultivars V (95%) vs H (5%) used in this study (Table 1). More importantly, the rapeseed genomic sequence was not released at that time. As such, the cultivars V and H served as ideal materials for RNA-seq to explore the candidate DEGs involved in secondary dormancy.

Analyses of all the indexes revealed that the RNA-seq data from three independent biological replicates in this study were of high quality and reliable (Additional file 2: Table S1). Based on a stringent threshold (|log_2_(FC) ≥ 1|and FDR ≤ 0.001), 998 candidate DEGs for secondary dormancy were identified as in V vs H, which were much more than those isolated in AC Excel vs DH12075 by *Arabidopsis* AR12K cDNA microarrays [7, 46]. Among the DEGs, those involved in “sulfur metabolism”, “tryptophan biosynthesis”, “glucosinolate metabolism” (Table 3) and “phytohormone signaling” (Additional file 1: Figure S7) were significantly enriched. GLSs, which are a type of sulfur-rich secondary metabolite composed of a β-D-glucopyranose residue linked via a sulfur atom to a (Z)-N-hydroximinosulfate ester plus a precursor amino acid-derived R group, are found mainly in Brassicaceae seeds [47, 48]. On the basis of the precursor amino acid and variable R group, GLSs can be classified into aliphatic (e.g., alanine (Ala)), indole (Trp) or aromatic (e.g., tyrosine (Tyr)) -GLSs [49]. Notably, different types of GLSs are synthesized by the enzymes of specific genes [48]. In this study, the expression level of specific genes for Trp-derived indole GLS biosynthesis were uniquely and -markedly upregulated in the dormant seeds (Fig. 3; Additional file 2: Table S8), while no changes were observed in the expression levels of the aliphatic or aromatic GLS biosynthesis genes (Additional file 2: Table S9). Correspondingly, the expression levels of the transcription factors *BnaA09g44500D* (*MYB51*), *BnaC02g22630D* (*MYB122*) and *BnaA05g18020D* (*MYC2*), which positively regulate indole GLS biosynthesis [50], were also upregulated (Additional file 1: Figure S10; Additional file 2: Table S10), while no changes were observed for the expression of *BnaCnng43220D* (*MYB28*) in the dormant seeds (Additional file 2: Table S10), which regulates aliphatic GLS biosynthesis. Notably, phenylpropanoid metabolism showed significant difference between PEG-treated AC Excel and DH12075 seeds [7], which is linked with GLS biosynthesis via aldoxime [23], suggesting that both GLS biosynthesis and related pathways are required for secondary dormancy.

Indole GLS is tightly linked to auxin biosynthesis [48]. Deficiency in GLS metabolism results in severe plant developmental defects that resemble auxin accumulation phenotypes [51–54]. Previous studies have demonstrated that auxin biosynthesis and signaling control primary dormancy in *Arabidopsis*, although some controversy remains to be solved. Genetic and molecular studies have revealed that auxin promotes primary dormancy by recruiting the auxin response factor *ARF10* and *ARF16* to control the expression of *ABI3* in *Arabidopsis* [23, 24]. In contrast to *ARF10* and *ARF16*, another auxin response factor, *ARF2*, is involved in seed germination by repressing the ABA signaling pathway [55]. Interestingly, the auxin influx carrier *AUX1*, which increases IAA levels, was recently reported to positively affect seed germination [26]. Recently, combined transcriptome and translatome analyses have revealed that Trp-dependent auxin biosynthesis inhibits *DOG1*-dependent primary dormancy in *Arabidopsis* [23]. In contrast to that conclusion, our data demonstrated that indole GLS-linked auxin biosynthesis increased in secondary seed dormancy (Figs. 3 and 4), and additional exogenous auxin treatment experiments provided evidence that auxin promoted secondary dormancy (Fig. 5), suggesting a discrepancy in primary vs secondary dormancy. As shown by Cadman et al. [56], transcriptomic analysis reveals there are substantial differences in gene expression genome-wide between primary and secondary dormant seeds in *Arabidopsis*. To our knowledge, this study is the first to provide evidence that auxin, acting as a versatile trigger in plant development, contributes to enhancing secondary dormancy in *Brassica napus*. However, how the auxin biosynthesis genes are activated remains unknown. In-depth studies on seed treatment applied for different lengths of time could provide insights into this regulation. Moreover, the mechanism by which secondary dormancy is induced by various stressful conditions differs from each other in *Arabidopsis* [56–58] and in barley [59–61]. It will be very interesting to reveal the specific and general mechanisms underlying secondary dormancy under different conditions in *Brassica napus*. Notably, the proportion of the explained phenotypic variation of the QTL using the population derived from a cross between AC Excel and DH12075 was somewhat lower, partly due to the less significantly phenotypic variation [16]. Therefore, a population will be constructed using cultivars V and H to detect QTL for secondary dormancy in our next work and it will be of great importance for molecular breeding and genomic research.

Extensive interaction between various hormones is required to determine the optimal developmental state for seed germination in various environments [62]. Notably, the exogenous IAA application doesn’t completely mimic secondary dormancy because of testa rupture and radical elongation (Fig. 5a and d), which suggested other phytohormones or factors are required in dormancy induction. In agreement, the MapMan overview of the regulation indicated that not only auxin but also other phytohormones are involved in secondary dormancy in *Brassica napus* (Table 5; Additional file 1: Figure S7). ABA level was significantly elevated in AC Excel secondary dormant seeds [7] and two out of four QTL confidence intervals for secondary dormancy partly overlap with those for ABA contents [16], suggesting ABA is a key factor in secondary dormncy. In agreement, the expression of the genes involved in ABA metabolism were significantly up-regulated in Va (Table 5). GA, which antagonistically functions with ABA, were down-regulated in Va (Table 5), further suggesting that the transcriptomic analysis was highly reliable. Surprisingly, another phytohormone, ethylene, which promotes seed germination, was found to be upregulated in dormant seeds (Table 5). Notably, a line of evidence has proved that auxin enhances ethylene production [63], which in turn specifically activates the indole GLS biosynthesis gene [64], suggesting an increase in ethylene may be a secondary reaction in response to auxin in secondary dormancy. Moreover, accumulating evidence has indicated that both brassinosteroid (BR) and auxin pathways synergistically control several auxin-responsive genes [65]. In this study, BR signalling components were upregulated in the dormant seeds. Although the role of jasmonic acid (JA) in seed dormancy is contradictory, JA specifically induces the expression of indole GLS biosynthesis genes [64], suggesting a synergistic effect on secondary dormancy with auxin. In addition to the aforementioned phytohormones, karrikins (KAR), the low-molecular-weight organic chemicals derived from the combustion of plant materials, have been shown to promote seed germination via elevated expression of a GA biosynthesis gene [66]. In the secondary dormant seeds, the expression of *KAI2*, the karrikin receptor, was significantly downregulated (Table 5). Together, all of these results suggest that the cross-talk between auxin and other phytohormones sophisticatedly regulates secondary seed dormancy in *Brassica napus*.

**Table 5:** Summary of DEGs involved in phytohormone biosynthesis and signalling transduction, with the exception of auxin

Hormone	Gene ID	*Arabidopsis* orthologs	Va vs Vb (log_2_FC)	Va vs Ha (log_2_FC)	Function description
ABA	BnaA04g16900D	CYP707A2	Down (−1.0)	Down (−1.9)	ABA degradation
	BnaC06g38870D	NCED9	Up (1.5)	Up (1.2)	ABA biosynthesis
	BnaA02g05170D	AAO1	Up (5.4)	Up (4.1)	ABA biosynthesis
GA	BnaA09g01090D	GA1	Down (−2.0)	Down (−2.6)	GA biosynthesis
	BnaA10g17240D	RGL3	Up (2.1)	Up (3.2)	GA negative regulator
	BnaAnng08970D	GASA1	Up (positive)	Up (positive)	GA response factor
ACC	BnaC09g13570D BnaA08g14220D	ACO2	Up (3.0/4.3)	Up (2.0/4.0)	Ethylene biosynthesis
	BnaA01g24090D	ETR2	Up (2.9)	Up (2.4)	Ethylene receptor
	BnaC01g10100D BnaAnng21280D	ERF1	Up (2.6/4.2)	Up (2.5/2.6)	Response to ethylene
	BnaC09g20090D BnaA06g35500D	ERF2	Up (2.3/2.4)	Up (2.9/1.3)	Response to ethylene
BR	BnaC07g47240D	BRI1	Up (2.4)	Up (2.3)	BR receptor
	BnaA08g00410D	BRL1	Up (2.9)	Up (2.0)	BRI1-like protein
	BnaC08g43180D	BSL2	Up (1.2)	Up (2.2)	BRI1 suppressor
	BnaA09g51800D	BSK3	Up (1.7)	Up (2.1)	BR signaling kinase 3
JA	BnaC06g26670D	LOX6	Up (1.8)	Up (1.2)	JA biosynthesis
	BnaA03g56600D	COI1	Up (1.4)	Up (2.6)	JA receptor
	BnaA06g13250D	JAZ1	Up (positive)	Up (positive)	JA signaling regulator
	BnaA10g20060D	JAZ10	Up (3.4)	Up (positive)	JA signaling regulator
	BnaA05g18020D	MYC2	Up (1.6)	Up (1.6)	JA signaling regulator
KAR	BnaA03g53980D	KAI2	Down (−1.7)	Down (−2.5)	KAR receptor

*ABA* abscisic acid, *GA* gibberellic, *ACC* ethylene, *BR* brassinolide, *JA* methyl jasmonate, *KAR* karrikin

In addition, the MapMan overview of cellular metabolism revealed that the DEGs are involved mainly in cell walls, lipid metabolism, secondary metabolism (Additional file 1: Figure S7a) and the regulation indicated the DEGs are enriched in transcription factors (Additional file 1: Figure S7b). Expression of cell wall-related genes, including *BnaA10g24090D* (*PGIP1*), *BnaA10g24090D* (*PGIP1*) [67] and *BnaC08g06930D* (*STOP1*) [68]; lipid metabolism genes, including *BnaA06g24040D* (*ACX2*) and *BnaC03g49190D* (*ACX2*) [69]; and secondary metabolism-related genes, including *BnaA08g21730D* (*CAT3*), *BnaC08g19360D* (*CAT3*) [69, 70] and *BnaA03g52000D* (*APX6*) [71], all of which were proved to be implicated in dormancy in *Arabidopsis*, exhibited significant changes in both Va vs Vb and Va vs Ha (Additional file 2: Table S3). In agreement, some of the DEGs involved in the pathways mentioned above was also identified in AC Excel vs DH12075 [7]. Several DEGs, including transcription factors, epigenetic modifiers and secondary metabolism genes were randomly selected for validation by qRT-PCR (Additional file 1: Figure S10), and the results generally matched well with the RNA-seq analysis.

## Conclusions

**Fig. 6:**
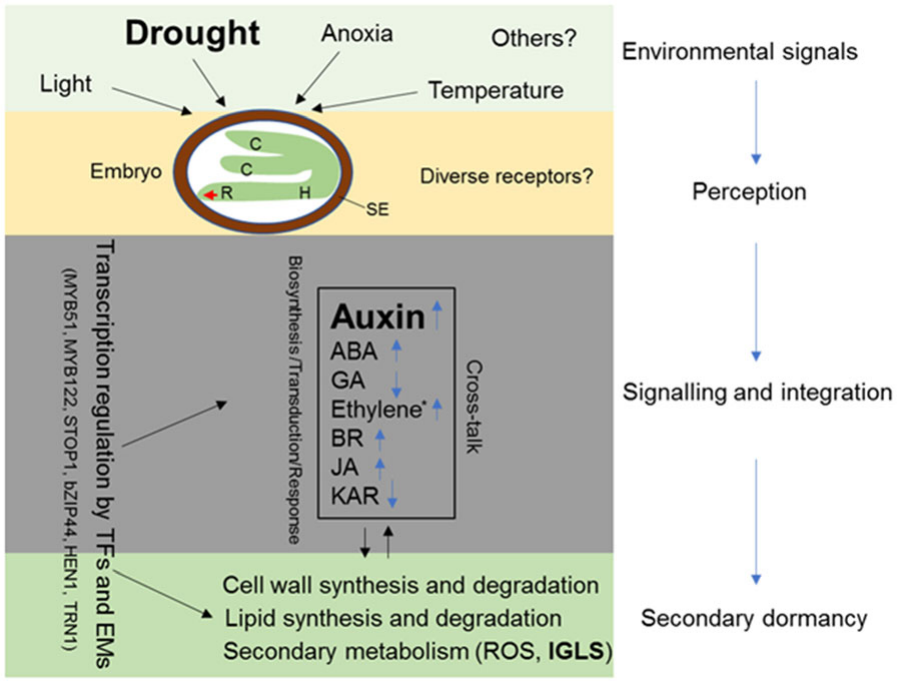
Schematic diagram for the mechanism underlying secondary seed dormancy induced by drought based on the DEGs in oilseed rape. Non-dormant rape seeds can be induced into secondary dormancy by adverse conditions (light, anoxia, temperature and drought) to keep alive to meet with the proper seasons for germination. The environmental signals are perceived by receptors in the embryo, possibly by diverse receptors. The signals are then transduced and eventually alter phytohormone signalling, including biosynthesis, transduction and responses. The phytohormone signals are integrated to regulate the expression of cell wall-, lipid- and secondary metabolism-related genes by transcription factors and epigenetic modifiers to induce and maintain secondary seed dormancy. PEG6000 is used as a proxy for drought in this study. C, cotyledon; H, hypocotyl; R, root; SE, seed envelope; ABA, abscisic acid; GA, gibberellic; BR, brassinolide; JA, methyl jasmonate; KAR, karrikin; TFs, transcription factors; Ems, epigenetic modifiers; IGLS, indole GLS

In this study, differentially expressed genes underlying secondary dormancy are explored by comparative transcriptome analysis using high secondary dormancy cultivar V and low secondary dormancy cultivar H. Our results showed that indole GLS-linked auxin biosynthesis genes are enriched and up-regulated in secondary dormant seeds (Va) compared with non-dormant ones (Ha) and subsequently confirmed by altered concentrations of indole-3-acetic acid (IAA), IAA conjugates and precursors. Based on our findings in this work, as well as those reported previously, a putative working model with respect to secondary dormancy induction was proposed (Fig. 6). In this model, stressful environmental cues are perceived by receptors in embryo and the signals are then transduced to alter various phytohormones biosynthesis and signaling, especially auxin biosynthesis genes are highly elevated (Fig. 3) in this study. The crosstalk among the phytohormone signaling is eventually integrated to regulate the cell wall-, lipid- and secondary metabolism-related genes by transcription factors and epigenetic modifiers (Additional file 1: Figure S10) to induce the secondary seed dormancy. This study reveals the important role of auxin in secondary dormancy in rapeseed, which will provide valuable information to decrease the soil seed bank.

## Additional files

Additional file 1: **Figure S1.** RNA quality was qualified by agarose gel electrophoresis (A) and RIN value (B). **Figure S2.** Distribution of read coverage against the reference genome of oilseed rape. **Figure S3.** Correlation analyses for each sample among three repetitions. **Figure S4.** Overview of the expression of genes in Va, Vb, Ha and Hb implicated in cellular metabolism (A) and regulation (B) pathways. **Figure S5.** Volcano plots for the total expressed genes among Ha vs Hb, Va vs Ha, Va vs Vb and Vb vs Hb. **Figure S6.** KEGG enrichment analysis corresponding to the secondary dormancy candidate DEGs. **Figure S7.** The MapMan overview of the cellular metabolism process (A) and regulation pathway (B) in which 998 DEGs are involved in. **Figure S8.** Germination assay with exogenous IAA application to Hb. **Figure S9.** Effects of ABA and IAA on secondary dormancy. **Figure S10.** Validation of 12 randomly selected DEGs via qRT-PCR. (DOCX 4606 kb)

Additional file 2: **Table S1.** Statistics of the RNA-seq results for the secondary dormancy of 12 samples. **Table S2.** Correlation analyses for each sample among three repetitions. **Table S3.** Detailed information of RNA-seq analysis. **Table S4.** DEGs isolated by the second round of selection based on the threshold |log2(Va vs Vb)-log2(Ha vs Hb)| ≥ 1. **Table S5.** Detailed GO enrichment analysis of the DEGs. **Table S6.** Detailed KEGG enrichment analysis of the DEGs. **Table S7.** Detailed list of all the DEGs corresponding to MapMan functional categories. **Table S8.** Detailed list of the DEGs involved in indole GLS-linked auxin biosynthesis. **Table S9.** List of genes involved in aliphatic and aromatic GLSs. **Table S10.** Differentially expressed transcription factors identified via RNA-seq analysis. **Table S11.** Differentially expressed epigenetic modifiers identified via RNA-seq analysis. **Table S12.** Seed quality differences in cultivars H and V. (XLSX 31002 kb)

## Data Availability

The dataset generated and analyzed during the study are available from the corresponding author on reasonable request.

## Abbreviations

ABA: Abscisic acid
GA: Gibberellic
GLS: Glucosinolate
IAA: Indole-3-acetic acid
IAA-Asp: IAA-aspartate
IAA-Glu: IAA-glutamate
IpyA: Indole-3-pyruvic acid
PEG: Polyethylene glycol
Trp: Tryptophan
