# Supplementary material for: A transcriptome analysis reveals a role for the indole GLS-linked auxin biosynthesis in secondary dormancy in rapeseed (Brassica napus L.)
Source: BMC Plant Biol. 2019 Jun 18;19:264. doi: 10.1186/s12870-019-1866-z (PMC6582522; doi:10.1186/s12870-019-1866-z)
Supplement: Supplementary file 1 — Figure S1. RNA quality was qualified by agarose gel electrophoresis (A) and RIN value (B). Figure S2. Distribution of read coverage against the reference genome of oilseed rape. Figure S3. Correlation analyses for each sample among three repetitions. Figure S4. Overview of the expression of genes in Va, Vb, Ha and Hb implicated in cellular metabolism (A) and regulation (B) pathways. Figure S5. Volcano plots for the total expressed genes among Ha vs Hb, Va vs Ha, Va vs Vb and Vb vs Hb. Figure S6. KEGG enrichment analysis corresponding to the secondary dormancy candidate DEGs. Figure S7. The MapMan overview of the cellular metabolism process (A) and regulation pathway (B) in which 998 DEGs are involved in. Figure S8. Germination assay with exogenous IAA application to Hb. Figure S9. Effects of ABA and IAA on secondary dormancy. Figure S10. Validation of 12 randomly selected DEGs via qRT-PCR. (DOCX 4606 kb) [file 12870_2019_1866_MOESM1_ESM.docx]

**Additional file 1**

**A transcriptome analysis reveals a role for the indole GLS-linked auxin biosynthesis in secondary dormancy in rape seed (*Brassica napus* L*.*)**

Lei Liu^1,2,#^, Fuxia Liu^1,2,#^, Jinfang Chu^4,#^, Xin Yi^1,2^, Wenqi Fan^1,2,3^, Tang Tang^1,2^, Guimin Chen^1,2^, Qiuhuan Guo^4^ and Xiangxiang Zhao^1,2,*^

^1^ Jiangsu Key laboratory for Eco-agriculture Biotechnology around Hongze Lake, Huaiyin Normal University, Huai’an 223300, China

^2^ Jiangsu Collaborative Innovation Center of Regional Modern Agriculture and Environment Protection, Huaiyin Normal University, Huai’an 223300, China

^3^ School of food science and engineering, Yangzhou University, Yangzhou 225127, China

^4^ National Centre for Plant Gene Research (Beijing), Institute of Genetics and Developmental Biology, Chinese Academy of Sciences, Beijing, China

^#^ These authors contributed equally to this work.

* Correspondence: [xxzhao2013@163.com](mailto:xxzhao2013@163.com);

Tel.:+86 517 83525885;

ORCID ID: 0000-0003-1392-3923


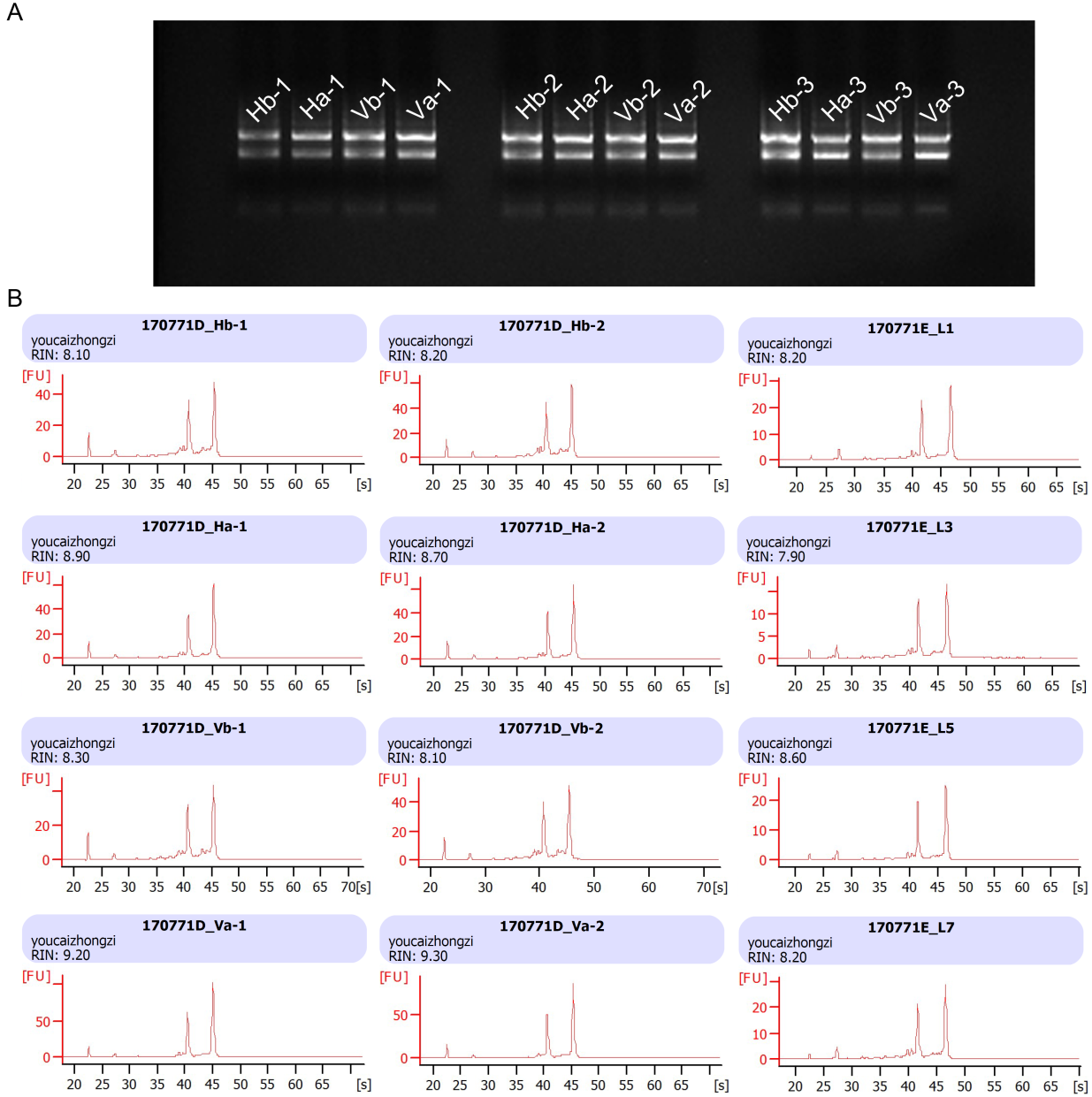


**Figure S1.** RNA quality was qualified by agarose gel electrophoresis and RIN value. (A) Total RNA isolated from mature seeds (Hb and Vb) and seeds subjected to prolonged imbibition in PEG in the dark (Ha and Va) (three independent replicates) is shown via agarose gel electrophoresis. (B) The RNA quality from each sample was further quantified via an Agilent 2100 Bioanalyzer. RIN values and electrophoresis for each sample are shown. The RIN value (≥7) was set as the threshold.


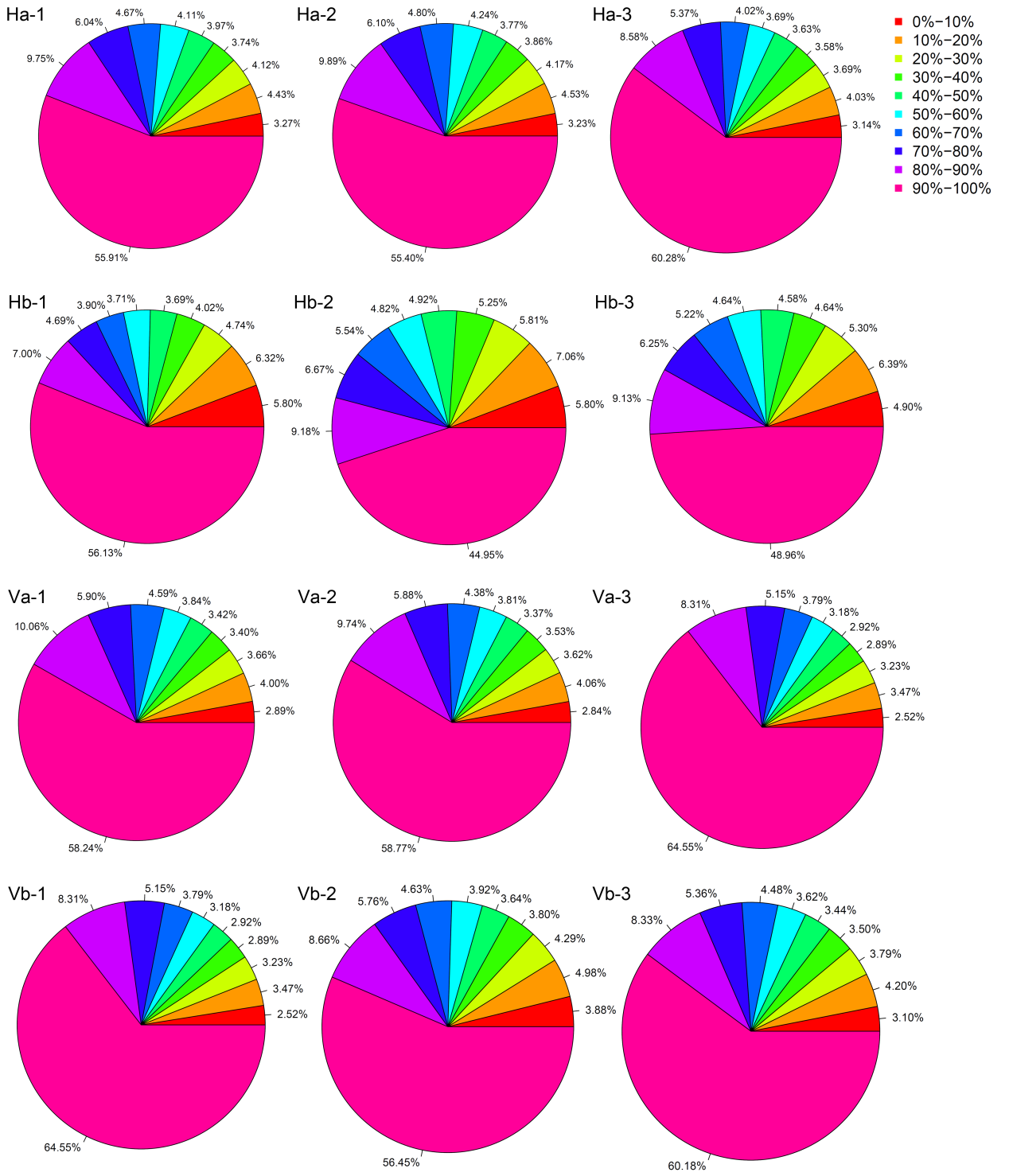


**Figure S2.** Distribution of read coverage against the reference genome of oilseed rape (*Brassica napus* L.).


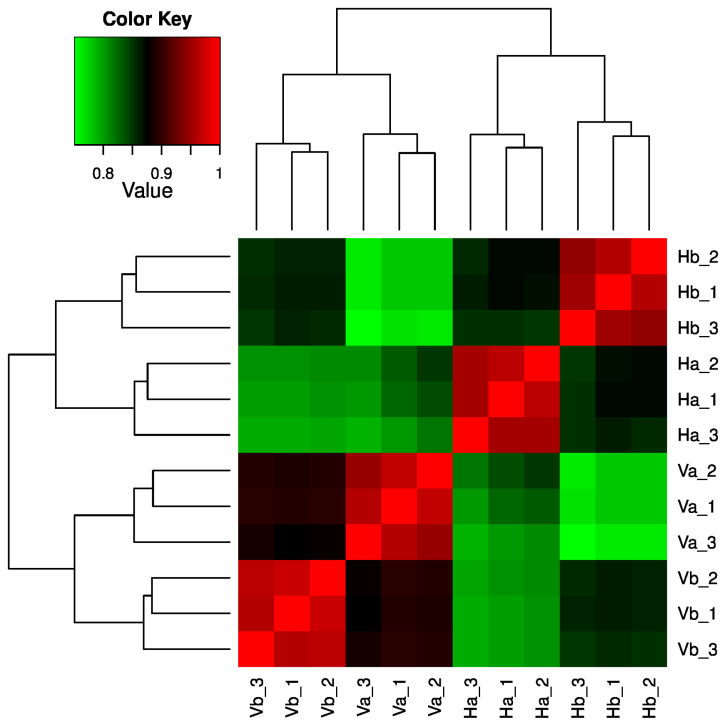


**Figure S3.** Correlation analyses for each sample among three repetitions.


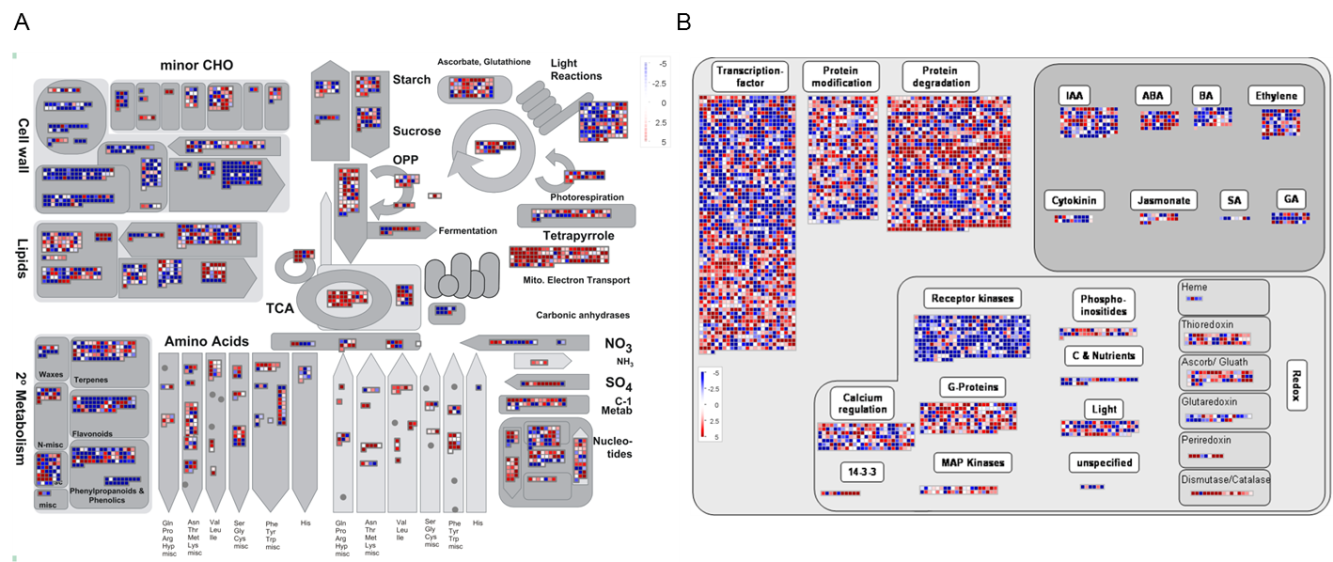


**Figure S4.** Overview of the expression of genes implicated in cellular metabolism (A) and regulation (B) pathways by MapMan software. Each box depicts an individual gene. The color bar from -5 (blue) to +5 (red) indicates the average FPKM normalized log2 transformed counts of each detected gene (i.e. log_2_(FPKM) value). The genes of which the log_2_(FPKM) value is greater than zero are shown in red and those of which the log_2_(FPKM) value is lower than zero are shown in blue, respectively.


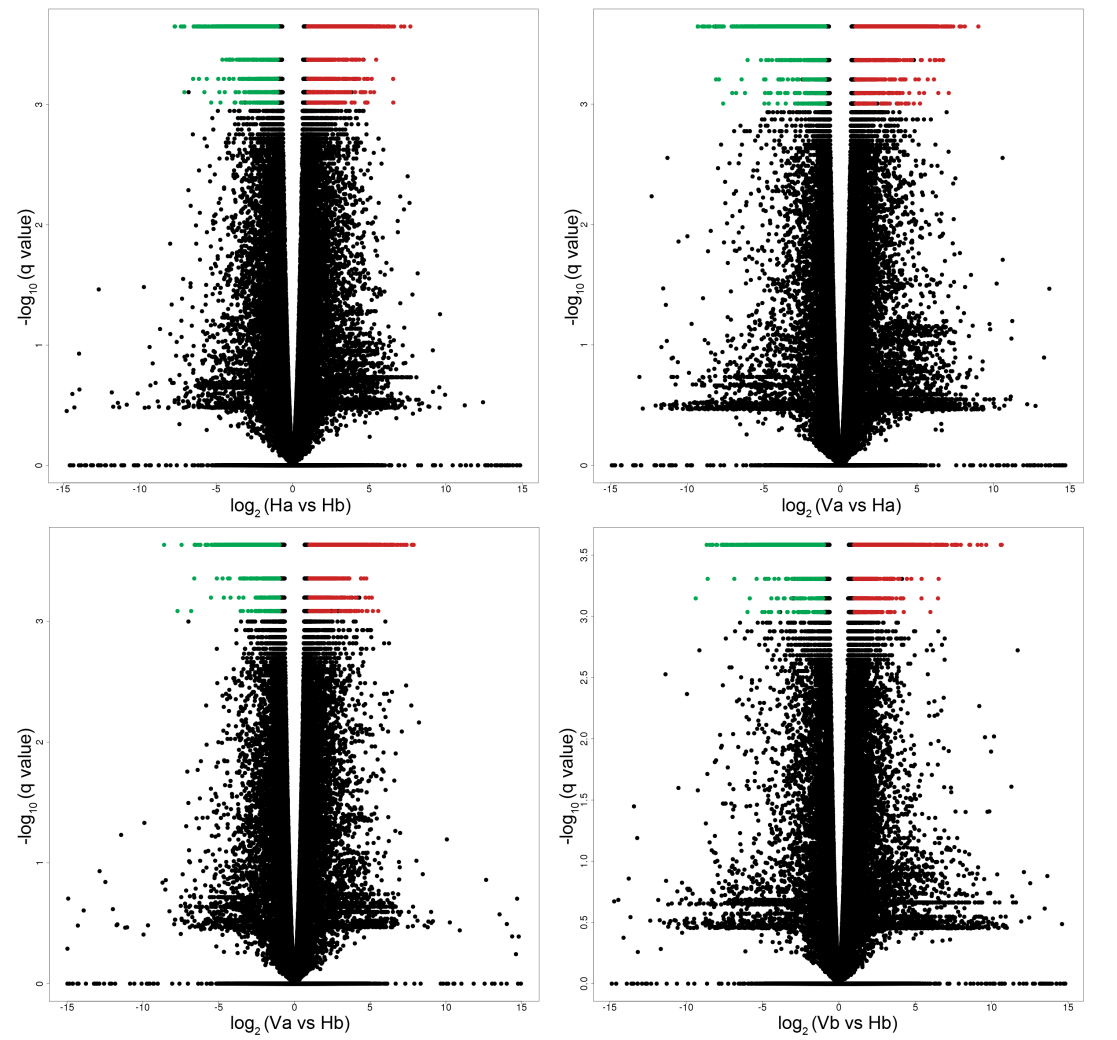


**Figure S5.** Volcano plots for the total expressed genes among the four comparison settings: Ha vs Hb, Va vs Ha, Va vs Vb and Vb vs Hb. The X- and Y-axes denote the log_2_(ratio) of the two samples and the -log_10_(q value), respectively. The red (upregulated) and green (downregulated) dots represent the genes whose expression was significantly different (|log_2_(ratio)| ≥1 and q value≤0.001), while the dark dots indicate the genes with no significant changes.


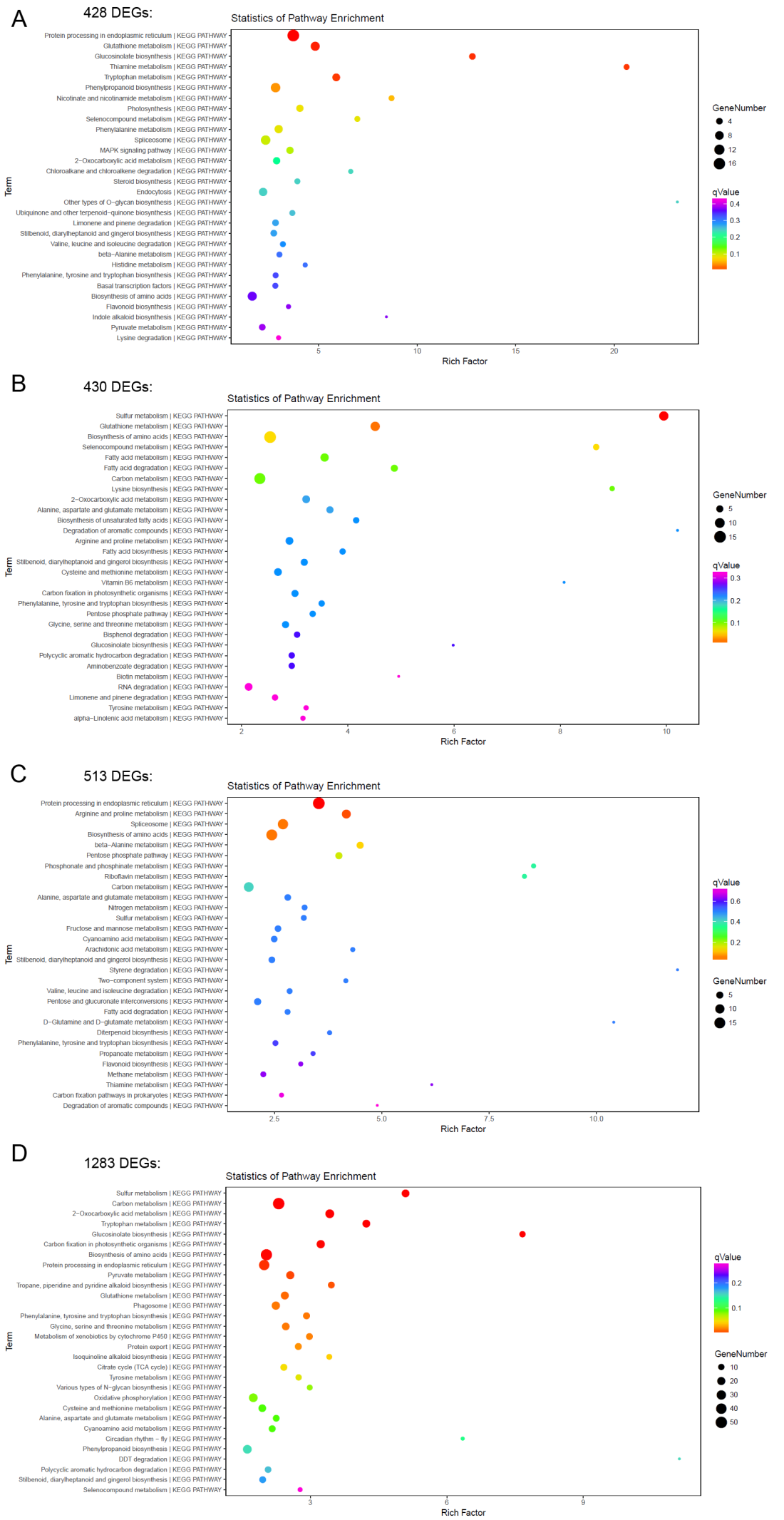


**Figure S6.** KEGG enrichment analysis corresponding to the secondary dormancy candidate DEGs in Fig. 2D in the intersection of all combinations (subgroup I) (A), the intersection of all combinations except Vb vs Hb (subgroup II) (B), the intersection of all combinations except Ha vs Hb (subgroup III) (C) and the specific interaction between Va vs Vb and Va vs Ha (subgroup IV) (D). The top 30 pathways are shown for representative subgroup.


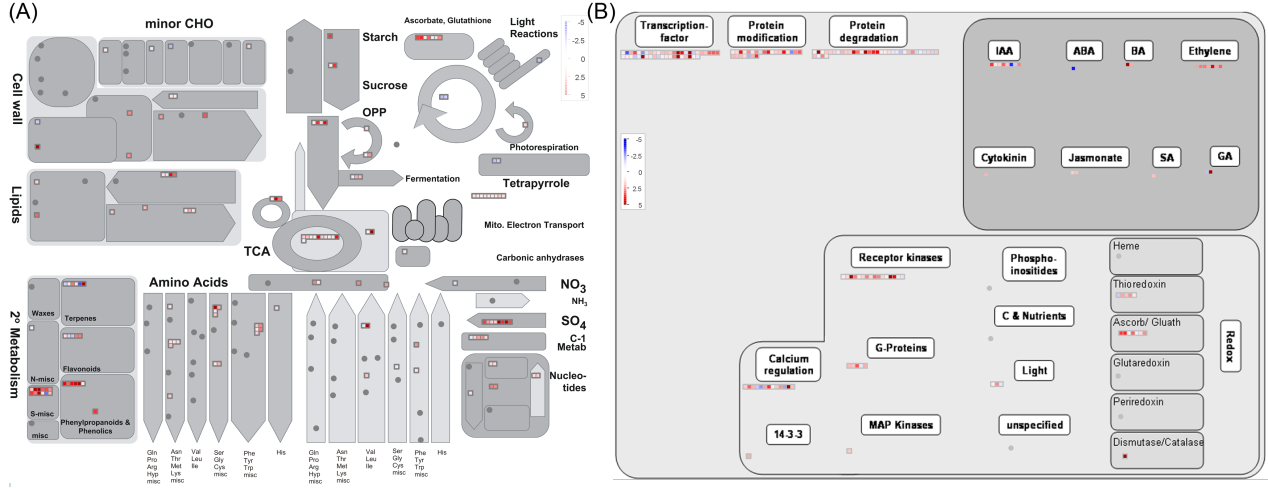


**Figure S7.** The MapMan overview of the cellular metabolism process (A) and regulation pathway (B) in which 998 DEGs responsible for secondary dormancy are involved in. Each box depicts an individual gene. The color bar from -5 (blue) to +5 (red) indicates the average counts of the log_2_(FC) between Va and Ha. The up- and downregulated genes are shown in red and blue, respectively.


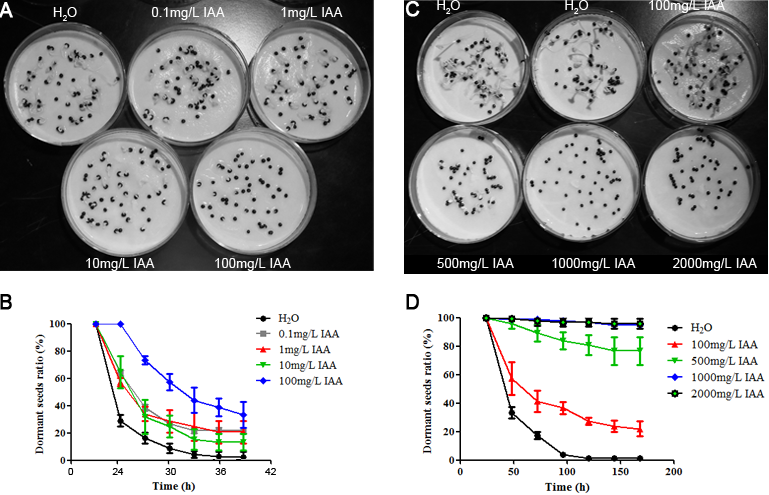


**Figure S8.** Germination assay with exogenous IAA application to Hb at 20°C in the dark. (A and B) Images and statistical analysis of germination supplemented with indicated exogenous IAA solution at the indicated time points. (C and D) Images and statistical analysis of germination supplemented with indicated exogenous IAA solution for 1 day and imbibition in water for another 7 days. The data were obtained from three independent replicates and are shown as means±SDs.


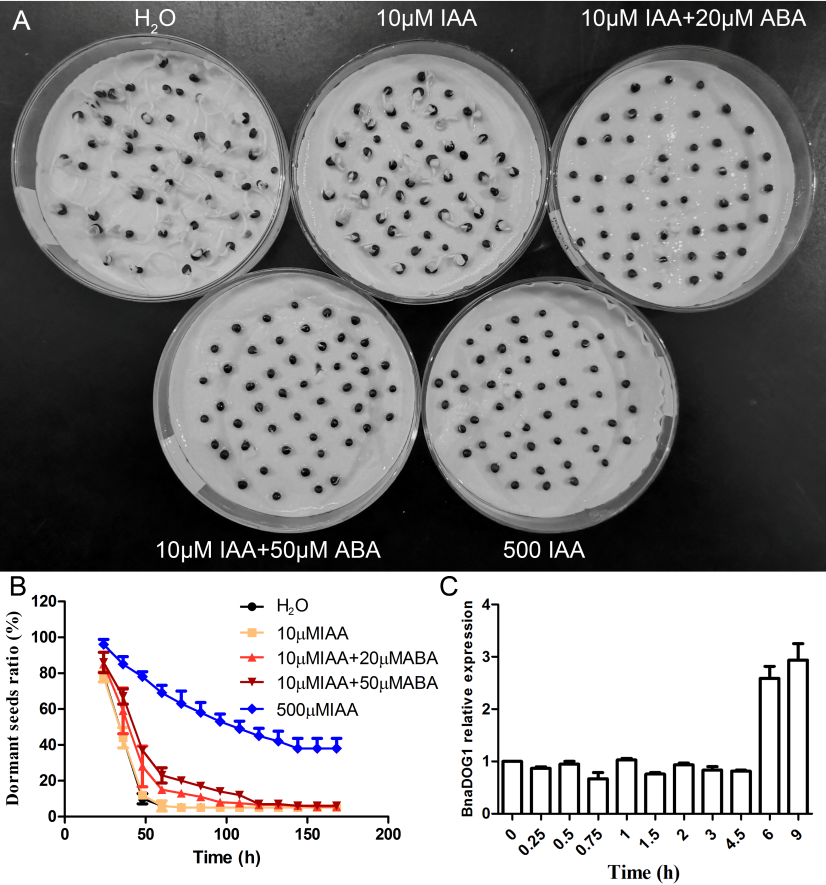


**Figure S9.** Effects of ABA and IAA on secondary dormancy. (A), Images of secondary dormancy ratio in various ABA and IAA concentrations. (B), Time courses of secondary dormancy rate in various ABA and IAA concentrations. (C), Time courses of *BnaDOG1* expression pattern in response to 100mg/L IAA.


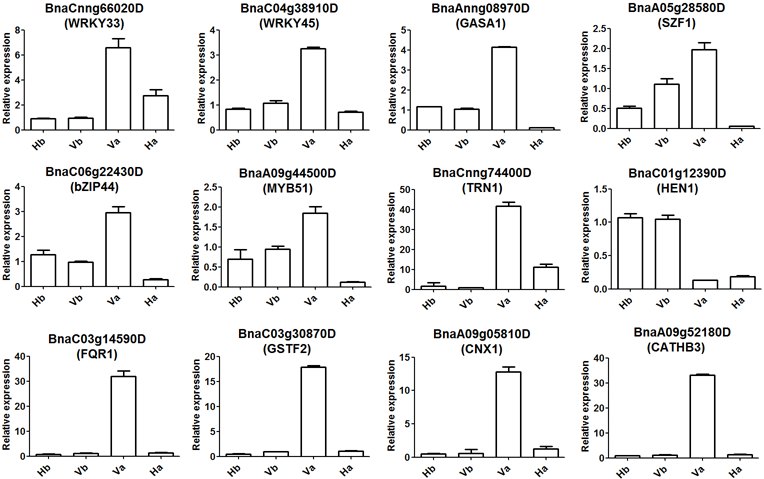


**Figure S10.** Validation of 12 randomly selected DEGs including transcription factors, epigenetic modifiers and secondary metabolism related genes via qRT-PCR. The relative mRNA levels of three biological replicates were calculated via the 2^-ΔΔCT^ method. The corresponding gene name is shown in the parentheses, as annotated by BLASTN in *Arabidopsis*. *BnaCAT1* was used as an internal control.
